# Structure-activity models of oral clearance, cytotoxicity, and LD50: a screen for promising anticancer compounds

**DOI:** 10.1186/1471-2210-8-12

**Published:** 2008-06-13

**Authors:** John C Boik, Robert A Newman

**Affiliations:** 1 Department of Experimental Therapeutics, University of Texas M. D. Anderson Cancer Center, 8000 El Rio, Houston, TX 77054, USA

## Abstract

**Background:**

Quantitative structure-activity relationship (QSAR) models have become popular tools to help identify promising lead compounds in anticancer drug development. Few QSAR studies have investigated multitask learning, however. Multitask learning is an approach that allows distinct but related data sets to be used in training. In this paper, a suite of three QSAR models is developed to identify compounds that are likely to (a) exhibit cytotoxic behavior against cancer cells, (b) exhibit high rat LD50 values (low systemic toxicity), and (c) exhibit low to modest human oral clearance (favorable pharmacokinetic characteristics). Models were constructed using Kernel Multitask Latent Analysis (KMLA), an approach that can effectively handle a large number of correlated data features, nonlinear relationships between features and responses, and multitask learning. Multitask learning is particularly useful when the number of available training records is small relative to the number of features, as was the case with the oral clearance data.

**Results:**

Multitask learning modestly but significantly improved the classification precision for the oral clearance model. For the cytotoxicity model, which was constructed using a large number of records, multitask learning did not affect precision but did reduce computation time. The models developed here were used to predict activities for 115,000 natural compounds. Hundreds of natural compounds, particularly in the anthraquinone and flavonoids groups, were predicted to be cytotoxic, have high LD50 values, and have low to moderate oral clearance.

**Conclusion:**

Multitask learning can be useful in some QSAR models. A suite of QSAR models was constructed and used to screen a large drug library for compounds likely to be cytotoxic to multiple cancer cell lines in vitro, have low systemic toxicity in rats, and have favorable pharmacokinetic properties in humans.

## Background

An ideal lead candidate for an anticancer drug is one that is non-toxic to the host, is well absorbed and so can be administered orally, and is effective at inhibiting cancer cell growth. Data on safety, pharmacokinetics, and cytotoxicity are expensive to generate in the laboratory, however, and there is need for developing reliable *in-silico *predictive models.

One aspect of developing reliable models is to make efficient use of all available training data. For example, if training data are available for an additional task that is related to the primary task of interest, such data could be useful in constructing a more reliable model. This paper explores the use of a multitask model for oral clearance, where bioavailability is the second task. It is among the first papers to report results for a human oral clearance model. Multitask models are most useful when data is limited, as is the case with the oral clearance model. In some cases, however, multitask models can also be useful with larger data sets. For the cytotoxicity models constructed here, use of a multitask model did not affect accuracy, but did reduce computation time (it reduced the computation time of the record selection algorithm).

Another aspect of developing reliable models is to base the models on random samples from well-defined populations. Moreover, the training populations should be very similar or identical to the population of compounds that one wishes to make predictions for. This is often difficult to achieve in practice, and new solutions are not proposed in this paper. This important topic is addressed more fully in the discussion section, but the reader should be aware that while the accuracy results presented here are valid for the training and testing sets, they may or may not be valid for predictions on other sets. Nevertheless, predictions are made here on other compounds to demonstrate the approach of using multiple QSAR models to screen a large compound library. Predicted results for any particular compound passing the screen would need to be verified in the laboratory. Such an approach has been used by Boik et al. [1] in a small study.

Predictive models of safety, pharmacokinetics, and cytotoxicity could be designed and used for a variety of purposes. Keeping in mind the model's limitations, the intended purpose in this paper was to screen a large library of natural products for those that might be suitable for preclinical study as components of anticancer drug mixtures. The criteria for suitability was that a compound be predicted to:

• inhibit multiple cancer cell lines in vitro at modest to low concentrations (IC50 of 50 *μ*M or below),

• be of low systemic toxicity (rat LD50 > 1920 mg/kg/day), and

• exhibit a low to modest oral clearance (<83 L/hr in humans).

Note that if the goal were to identify promising compounds for study as individual drugs, as opposed to components of mixtures, different criteria would likely be used. For example, more potent cytotoxic agents might be desired. In addition, only novel compounds might be of interest.

Three QSAR classification models were constructed. QSAR models identify statistical relationships between a response (also called a task or target) and molecular features of a compound, such as molecular weight, logP, and functional group counts. As noted above, the three proposed models are based on data for human oral clearance, rat LD50, and *in-vitro *cytotoxicity. Oral clearance is a measure of the rate of drug removal from the body after oral administration, and LD50 refers to the expected dose needed to kill 50 percent of an animal population. The three models were applied to a set of over 115,000 natural compounds and hundreds were predicted to be cytotoxic, of low systemic toxicity, and of low to modest oral clearance.

The data modeled here were challenging. Correlations between single features and responses were very weak and many of the features were highly correlated with one another. In addition, a large number of features (>1600) were employed, which in the case of the oral clearance model was greater than the number of records. (The term *record *is used to refer to the combination of observed responses and calculated features for a single drug.) Lastly, the processes modeled were biochemically complex and observed responses were noisy. For example, measurements of oral clearance commonly exhibit a within-study coefficient of variation of 25 to 100 percent [2-6]. In a 1979 report, LD50 values were observed to vary by as much as 3- to 11-fold between different laboratories [7]. *In-vitro *cytotoxicity data were also noisy, typical of high-throughput screening experiments. With regards to classification, noisy measurements are particularly problematic when they occur at the thresholds used to demarcate active from inactive drugs. In the cytotoxicity data modeled here, compounds were concentrated near these thresholds.

Models were constructed using Kernel Multitask Latent Analysis (KMLA), an algorithm developed by Xiang and Bennett [8] based on earlier work by Momma and Bennett [9] and used here with minor changes. KMLA is closely related to partial least squares (PLS), an algorithm that is commonly used in QSAR and microarray studies when features are highly correlated and the number of records is small compared to the number of features [10-16]. PLS algorithms were originated by Wold in the 1970s and were later refined by a number of researchers [17-20]. Briefly, PLS algorithms use a series of linear projections to create a small set of orthogonal "latent" data columns from the original data so that the covariance between the latent features and response is maximized. In this way, the dimensions of the original data are greatly reduced, problems with correlated features are eliminated, and maximal information related to the response is retained. Whereas PLS produces linear models, KMLA can produce nonlinear models, with the degree of nonlinearity determined by the choice of kernel function and kernel parameters.

KMLA is designed for (nonlinear) multitask learning. Multitask learning can be useful when the number of records is small relative to the number of features [21], as is the case for the oral clearance data. Multitask learning models have been proposed by several authors [22-25], although their use in QSAR is still rare. In KMLA, collective learning is ensured by forcing all problems to use a shared set of latent features. This type of collective learning has been referred to as *common feature mapping*. In the common latent feature space each task is independently treated as a single-task learning problem [26]. Because each task is independently modeled, tasks need not share common records and multiple types of models can be used (classification for one task and regression for another, for example). The common set of latent features is obtained by minimizing loss functions across modeled tasks.

## Results

### Oral clearance

Oral clearance can be calculated as clearance divided by bioavailability, where clearance refers to the systemic clearance after intravenous administration (in units of volume/time) and bioavailability is a fraction less than or equal to 1.0. Because there is an upper limit to the systemic clearance (based on physical constraints), very high oral clearance values are associated with very low bioavailability. High oral clearance is, in general, not a desirable characteristic for compounds that are being developed as oral drugs.

The complexities of physical events that impact oral clearance make accurate prediction of oral clearance difficult to achieve. Not surprisingly, the accuracy of predictions made by the oral clearance model was not high under any model variation tested here. Accuracy improved, however, when multitask learning was used, with bioavailability as an additional task. By definition, bioavailability is related to oral clearance and the relationship should be particularly strong for drugs with high oral clearance values.

Results are summarized in Table 1 for models that employed three latent features. The five oral clearance models are given the abbreviations OC.1 through OC.5 (OC for oral clearance). Models were replicated 32 times and standard deviations of results are given in parentheses.

**Table 1:** Results for oral clearance (OC) models

**Model**	**Task**	**Kernel**	**Features**	**Average test set precision, (+) labels**	**Average test set precision, (-) labels**
OC.1	oral clearance	Gaussian	subset	0.649 (0.060)	0.569 (0.111)
OC.2	oral clearance	linear	subset	0.606 (0.071)*	0.547 (0.107)**
OC.3	oral clearance & bioavailability	Gaussian	subset	0.636 (0.060)	0.625 (0.091)
OC.4	oral clearance & bioavailability	Gaussian	all features	0.626 (0.066)	0.634 (0.108)***
OC.5	oral clearance & bioavailability	linear	subset	0.635 (0.061)	0.637 (0.093)

* Significant differences by ANOVA (p < 0.05): OC.1 vs. OC.2

** Significant differences by ANOVA (p < 0.05): OC.2 vs. OC.3, OC.4, and OC.5 when testing OC.1 through OC.5

*** Significant differences by ANOVA (p < 0.05): OC.4 vs. OC.1 when testing OC.1, OC.3, and OC.4

The last two columns of the table provide test set precision for positive and negative labels averaged over all replications. Precision on the positive labels is defined as the number of records that are true positives and predicted to be positive, divided by the total number of true positives. Precision on the negative labels is defined as the number of records that are true negatives and predicted to be negative, divided by the total number of true negatives.

Confusion matrices can be constructed from the data given in the Table 1. The two column labels of the confusion matrix are *true positives *and *true negatives*, respectively. The two row labels are *predicted positive *and *predicted negative*, respectively. The diagonal elements of the confusion matrix, expressed as fractions, are given in the last two columns of Table 1. Using model OC.1 as an example, the diagonal of the confusion matrix would be 0.649 and 0.569. The lower left cell would be 1.0–0.649 and the upper right cell would be 1.0–0.569. There were 435 oral clearance records in total and about 20 percent (87) were used for testing in any given replication. Of these, about 70 percent (61) were true positives and about 30 percent (26) were true negatives (see Table S.1 in Additional File 1). Thus for example, the diagonals of the confusion matrix for OC.1 would be about (0.649)(61) and (0.569)(26), respectively, if expressed in the units of records.

Single task models and linear models tended to preform worse than multitask models and gaussian kernel models. When precision on negative labels was compared for all models, precision for OC.2 (a single-task model) was significantly lower than that for all others (ANOVA with family-wise error correction, *p *< 0.05, see Table 1). When precision on negative labels was compared only for Gaussian models, precision for OC.4 (a multitask model) was significantly higher than that for OC.1 (a single-task model). When comparing only the single task models, precision for the Gaussian model was significantly higher than that for the linear one.

The training algorithm was designed to maximize total precision and minimize the difference in precision between the positive and negative labels. The percentages of positive and negative labels in the complete data set were unequal, however, which generally makes balanced precision more difficult to achieve. Indeed, test set precision for the single task models (OC.1 and OC.2) was lower for the negative labels than for the positive labels (the larger of the two groups). Precision for the multitask models was more balanced, however.

For all model variations tested, use of model averaging tended to improve precision. Table S.2 in Additional File 1 lists results using OC.1 and OC.3 as examples. Although the trend was consistent across all models, differences were not significant (compare, for example, the values of 0.649 and 0.623 for the positive labels in the second column of the table).

To further investigate model validity, Model OC.3 was estimated again using scrambled response values. Average test-set precision for positive and negative labels was 0.494 (stdev = 0.057, *n *= 8) and 0.565 (stdev = 0.056, *n *= 8), respectively, which was significantly different from the means of 0.636 and 0.625 obtained for OC.3 when labels were not scrambled (*t*-test, *p *= 3.4E-07 and *p *= 0.024, respectively). The expected results for scrambled records would have been 0.5 for both positive and negative labels if model averaging had not been used. With model averaging, there was a small bias towards negative predictions and this bias had a relatively larger impact on the precision for the negative labels (the smaller of the two groups).

To verify that the KMLA algorithm provided some benefit over a standard partial least squares algorithm, an oral clearance model was estimated using the generalized partial least squares (gpls) library of R [27]. To make the comparison as fair as possible, KMLA was used in single-task mode, paired training and verification sets were used between approaches, three latent variables were used for each approach, and hyper-parameter selection (based on validation set results) was done in the same manner for each approach. Eight replicate models were constructed and results were assessed using the one-sided paired *t*-test. In comparing the KMLA and gpls approaches for single task models, KMLA has the advantages that model averaging and cost-sensitive learning can be used. In addition, nonlinear responses can be modeled (the kernel used here was near-linear, however). Without the use of model averaging, precision on the negative labels (the more challenging of the two classes) was higher for the KMLA algorithm (0.60 vs. 0.57) but the difference was not statistically significant. When model averaging was used, precision was again higher for the KMLA algorithm and the difference was statistically significant (0.67 vs. 0.57, *p *= 0.025). Differences in precision for the positive labels were not statistically significant.

In summary, use of a multitask model modestly but significantly improved precision on the negative labels. Multitask models also tended to exhibit more balanced precision between positive and negative labels. Use of model averaging also tended to improve precision, although differences were not statistically significant. The KMLA algorithm preformed slightly better on an oral clearance task than did a standard partial least squares algorithm.

### Rat LD50

The number of records in the rat LD50 data set (3,869) was more than twice as large as the number of features, yet it was small enough that all records could be modeled in a reasonable amount of time. Results are summarized in Table 2 for models that employed four latent features. The two models are referred to as LD.1 and LD.2 (LD for lethal dose). Models were replicated 8 times and standard deviations are given in parentheses. This was a lower number of replicates than used for the oral clearance model, as the LD50 data set was larger and modeling results were less variable. The LD50 data set contained only one response and so KMLA was used in the single-task mode.

**Table 2:** Results for rat LD50 (LD) models

**Model**	**Kernel**	**Average test set precision, (+) labels**	**Average test set precision, (-) labels**
LD.1	linear	0.702 (0.042)	0.674 (0.021)*
LD.2	Gaussian	0.717 (0.029)	0.707 (0.019)

* Significant differences by ANOVA (p < 0.05): Model 1 vs. Model 2

Model LD.2, which used a Gaussian kernel, performed significantly better on the negative labels (the larger of the two classes) than the model using a linear kernel (ANOVA with family-wise error correction, *p *< 0.05, see Table 2). Model averaging tended to improve precision but differences were not significant. Without model averaging, average test-set precision for the positive and negative labels of LD.2 was 0.703 and 0.699, respectively.

Confusion matrices can be constructed from the data given in Table 2. There were 3,869 records in total, about 20 percent of which (774) were used for testing in any given replication. Of these, about 33 percent (255) were true positives and about 67 percent (511) were true negatives (see Table S.1 in Additional File 1).

### Cytotoxicity

The number of records in the cytotoxicity data set (8,983) was more than five times as large as the number of features. It was not practical to model more than about 4,500 records, however, and a subset of records was used. Only about five percent of records had a positive label, and so all of these were retained for modeling along with a selected group of records with negative labels. Multitask models were constructed using LC50 and TGI (total growth inhibition) responses; models were desired for both responses and it was expected that the biochemical processes involved in both were related. Results are summarized in Table 3 for models that employed three latent features. The eight models are referred to as C.1 through C.8 (C for cytotoxicity). Models were replicated 8 times and standard deviations are given in parentheses. Testing sets contained about 2,695 records.

**Table 3:** Results for cytotoxicity (C) models

**Model**	**Cell Line**	**Task set**	**Kernel**	**Average test set precision, (+) labels**	**Average test set precision, (-) labels**
C.1	H460	LC50	Gaussian	0.707 (0.032)	0.809 (0.008)
C.2	H460	LC50 & TGI	Gaussian	LC50: 0.711 (0.034) TGI: 0.732 (0.021)	LC50: 0.809 (0.009) TGI: 0.834 (0.009)
C.3	H460	TGI	Gaussian	0.729 (0.028)	0.838 (0.009)
C.4	H460	LC50	linear	0.630 (0.056)*	0.788 (0.014)*
C.5	H460	TGI	linear	0.683 (0.047)**	0.803 (0.013)**
C.6	MCF7	LC50	Gaussian	0.675 (0.019)	0.801 (0.011)
C.7	MCF7	LC50 & TGI	Gaussian	LC50: 0.674 (0.036) TGI: 0.685 (0.027)	LC50: 0.798 (0.016) TGI: 0.820 (0.012)
C.8	SF-268	LC50 & TGI	Gaussian	LC50: 0.665 (0.056) TGI: 0.698 (0.047)	LC50: 0.826 (0.014) TGI: 0.845 (0.013)

* Significant differences by ANOVA (p < 0.05): C.4 vs. C.1 and C.2

** Significant differences by ANOVA (p < 0.05): C.5 vs. C.2 and C.3

The Gaussian kernels performed significantly better than the linear kernels for the H460 cell line for both positive and negative labels (ANOVA with family-wise error correction, *p *< 0.05, see Table 3). Based on these results, only Gaussian kernels were tested for the other cell lines. The multitask models did not perform significantly better than the single-task models, as expected given the large number of training records. Multitask models did reduce training time, however. In particular, the time needed to select records for inclusion in training sets was cut in half. Confusion matrices can be constructed from the data given in Table 3. The number of records in each cytotoxicity data set is given in Table S.3 in Additional File 1.

Model averaging consistently tended to improve precision, although differences were not significant. For example, without model averaging, average test-set precision for positive and negative labels for C.2 (LC50) were 0.703 and 0.801, respectively, as compared to 0.711 and 0.809, respectively, with model averaging.

The models listed in Table 3 were based on a subset of records, where the subset was chosen using an algorithm described in the Methods section. As an alternative, records with negative labels could be randomly chosen. Using Model C.2 (LC50) as an example, the randomization method reduced the precision for negative labels from 0.809 to 0.698. The precision for positive labels was not greatly changed (0.724 vs 0.711), which was expected because the same set of positive labels was used in both models. The inferior performance of the model with random negative labels is due to the fact that some labels useful for classification were left out. In contrast, selection of negative labels by the algorithm helped assure that useful records were retained.

Precision of a final cytotoxicity model was investigated in three ways, using Model C.2 (LC50) as an example. First, the precision was determined for the 1,626 compounds that were contained in both the NCI data set and the set of 115,000 natural compounds. Some of these duplicate compounds would have been included in the training set for C.2 and some (with a negative label) would have been excluded from it. Of the 1,626 compounds, 148 had a positive label and 1,478 had a negative one. The precision was 0.74 and 0.73, respectively.

Second, the precision was determined for a smaller set of 557 compounds that were contained in the natural compound set and for which additional NCI cytotoxicity data were available. Most of these were compounds that were added to the NCI database after 2005. None of them was used in training the model. There were 83 compounds with positive labels and 472 with negative ones. Precision was 0.48 and 0.72, respectively. Given that the testing set precision on the positive labels was much higher (0.71), and that the test sets contained far more compounds (about 2,700), it seems likely that the lower precision on the new data may have been due to differences in sample composition. Neither the set of 557 nor the set of 1,626 mentioned above was a random sample from the natural compounds set.

Third, precision was determined for the set of 557 compounds as above, only using a model that was constructed with scrambled training responses. Average precision on the positive and negative labels was 0.25 and 0.51, respectively (*n *= 2). The low precision on the positive labels was due to the low percentage of positive labels in the training set.

In summary, a model with Gaussian kernel performed significantly better in cross-validation than one with a linear kernel for the H460 cell line, and use of a multitask model reduced the time needed for record selection. Model averaging tended to improve precision, and the algorithm for choosing negative training records was more useful than random selection.

### Predictions for natural compounds

Models described above were used to make predictions for a set of more than 115,000 natural compounds. Gaussian kernels were employed, and multitask models were used for cytotoxicity and oral clearance.

The number and fraction of natural compounds passing various screening criteria are listed in Table 4. Compounds passed a given screen if their predictions were all positive for the selected criterion. Screens 5 to 8 emphasize the H460 model for illustrative purposes (any of the three cells lines could have been used). The purpose of the first, most rigorous screen is to identify compounds that are likely to have low acute toxicity, possess cytotoxic activity, and be suitable for oral administration. These compounds would be a priority for preclinical study, where their properties could be confirmed.

**Table 4:** Summary of screening results

**Screen**	**Screening criteria**	**Fraction passing**	**Number passing**
1	Oral clearance, LD50, and all six cytotoxicity models	0.0036	416
2	Oral clearance, LD50, and passing in both H460 models and both LC50 and TGI of either MCF7 or SF-268 models	0.0043	498
3	LD50 and passing in both H460 models and both LC50 and TGI of either MCF7 or SF-268 models	0.035	4,014
4	Oral clearance and passing in both H460 models and both LC50 and TGI of either MCF7 or SF-268 models	0.017	1,981
5	Oral clearance, LD50, and passing in both H460 models	0.0045	520
6	Oral clearance and passing in both H460 models	0.039	4,458
7	LD50 and passing in both H460 models	0.022	2,255
8	Passing both H460 models only	0.24	27,608

Comparing results from Screens 1, 2, and 5, compounds that were predicted to be active in the H460 cytotoxicity models also tended to be predicted active in the other two cell lines. Comparing Screens 7 and 8, most of the compounds that were predicted to be active against the H460 cell line also were predicted to be toxic to rats. Indeed, Halle [28] reported that *in-vitro *cytotoxicity can be used to predict rat LD50 values. Not surprisingly, given a prediction of cytotoxicity the criterion of passing the LD50 model was more restrictive than that for passing the oral clearance model (compare Screens 6 and 7).

The diversity of molecules passing the more restrictive screens was considerably lower than the diversity of molecules in the entire natural compounds data set. For molecules passing the more restrictive screens, two groups were heavily represented: anthraquinones and flavonoids. A typical molecule from the first group was aloe-emodin, and ones from the second group were quercetin glycosides.

In summary, compounds that were predicted to be cytotoxic in one cell line were usually predicted to be cytotoxic in the other cell lines, as well as systemically toxic to rats. As would be expected, use of the cytotoxicity and LD50 screens together resulted in far fewer passing compounds compared to use of the cytotoxicity screen alone or use of cytotoxicity and oral clearance screens. Many of the compounds that passed the more restrictive screens were either anthraquinones or flavonoids.

## Discussion

### Multitask learning

QSAR models in biology often suffer from a lack of available records for training and testing. This was the case with the oral clearance model and the results presented here suggest that for this model, multitask learning can modestly but significantly improve precision (see Table 1). Multitask learning did not significantly improve precision of the cytotoxicity models (see Table 3), but this result was expected due to the large number of records available for training. However, multitask learning did reduce computation time for the combined LC50 and TGI models (it reduced the time for record selection by half). Multitask learning has not yet become popular for QSAR modeling, and these results suggest that it could play a larger role. For the tasks modeled here, the results also show that nonlinear models can in some cases perform significantly better than linear ones. Model averaging also tended to improved accuracy, but differences were not significant.

### Comparison with published models

The models developed here seem to be of comparable accuracy to ones previously published in the literature, however such comparisons are difficult to make because each published study used different data and a different modeling approach. Ralaivola et al. [29] and Swamidass et al. [30] used graph kernels to construct QSAR models of cytotoxicity, also based on the NCI data set. Their approach differed in that they modeled GI50 rather than LC50 or TGI values. GI50 is a measure of the growth inhibitory power of a compound, TGI is a measure of cytostatic effect, and LC50 is a measure of cytotoxic effect. By design, GI50 < TGI < LC50. The record selection methods and threshold values they used resulted in training sets that were nearly balanced between positive and negative labels. In comparison, the average fraction of positive labels in the NCI data modeled here was only 0.05. One could expect better predictive accuracy under more balanced conditions. Even so, results presented in Table 3 were comparable. For the three cell lines modeled here, Ralaivola et al. [29] reported an average precision of 0.74, whereas average precision for TGI here was 0.71. By modeling LC50 and TGI rather than GI50, the models developed here are designed to identify compounds with higher average potency.

Several QSAR models of human oral absorption [31-39] and bioavailability [40-43] have been published for heterogenous sets of compounds, but papers on oral clearance are rare. In comparing the complexity of the physiological events involved in absorption, bioavailability, and oral clearance, one would expect that oral absorption models would be the most accurate of the three and oral clearance models would be the least accurate. For example, first-pass metabolism is not accounted for when measuring absorption, and systemic clearance by the liver is not accounted for when measuring bioavailability. Indeed, Hou et al. [44] reported that human bioavailability was much more difficult to predict than oral absorption. In a multi-label classification study of bioavailability based on 432 drugs, Pintore et al. [40] reported that average test set accuracy was 0.75 (fraction of correctly classified compounds) over all classes. Yoshida and Topliss [43] published a multi-label classification study on bioavailability of 232 drugs and obtained a somewhat lower average test set accuracy (0.60). This result for bioavailability is similar to the one reported here for oral clearance (average precision of 0.63 across positive and negative labels).

Only one QSAR study on oral clearance could be found. Wajima et al. [45] published a regression model for oral clearance that used 87 drugs and produced a cross-validation *q*^2 ^correlation coefficient of 0.694. Some of the features were generated from animal pharmacokinetic experiments, however, and not from chemical structure alone as done here. It can be expected that human oral clearance would be more correlated with animal pharmacokinetic data than with molecular descriptors.

Numerous QSAR models of rat LD50 have been published, but almost all of these used smaller, homogenous sets of compounds [46-51]. Commercial QSAR LD50 models (e.g., TOPKAT [52] and MCASE [53]) also tend to use multiple QSARs on smaller, homogenous sets of data. In a comparison of several commercial QSAR models, Tunkel et al. [54] reported that 67 percent of multi-label predictions by TOPKAT were correct (based on 73 chemicals unseen by the model), and 70 percent of predictions by MCASE were correct. Neither model was able to classify all 73 compounds because some were outside the training sets. While the LD50 model developed in this paper is not multi-label, it did correctly classify 71 percent of compounds in the test sets.

Of the three models constructed here, the oral clearance model was the least accurate (precision of 0.636 and 0.625 for positive and negative labels, respectively, for model OC.3). Nevertheless, the model is still of interest for two reasons. First, this is one of the few published attempts at modeling oral clearance and better results have not been reported for comparable data. Second, the model variants that were constructed suggest that use of multitask learning may impart a small but significant learning advantage.

### Model generalizability

In this paper, three classification models were constructed for oral clearance, cytotoxicity, and rat LD50 data, respectively, and predictions were made for a large set of natural compounds. In all three models, the largest publically available data sets were employed (with some compounds failing the inclusion criteria). Each set of compounds used was a sample from some larger population of compounds, but it seems highly unlikely that they were random samples. Moreover, the set of natural compounds is unlikely to represent a random sample from the population of all natural compounds that could pass the inclusion criteria. Furthermore, the training sets were unlikely to be random samples from the natural compounds population.

The populations that the training sets were drawn from are essentially unknowable, and therefore true random samples from those populations cannot be collected. For example, oral clearance values were taken from the literature and may have been subject to publication bias. One might expect published results to be rich in compounds that have some significance to pharmacology or toxicology, and that have low to modest oral clearance. The cytotoxicity data provide another example. NCI selected particular compounds for screening based on a variety of concerns, which likely included expected activity, chemical structure, past results, and the submissions made to the NCI program.

In spite of the uncertainties of the populations, these are the best available public data on which to base models and so are used here. There is some assurance that the models will generalize to the hold-out test sets, as demonstrated by cross-validation, but there is little assurance that the models will generalize to any new set of compounds collected by NCI or others, or to the set of natural compounds on which predictions were made. One would hope, however, that the size of the data sets, particularly the NCI and LD50 data sets, might increase the generalizability of the models.

The best way to test generalizability would be to know the populations from which the training sets were taken and then to randomly sample compounds from that population for additional testing. If these populations were known, a sizable sample of compounds, perhaps many hundreds, would need to be tested in the laboratory. Such an undertaking is beyond the scope of this project. Furthermore, this still would not address the issue that the prediction set of natural compounds may be different from the training sets. If the only population of interest were the particular set of natural compounds used here, a random sample from that set could be tested in the laboratory. But again the sample would need to be large.

In spite of the uncertain generalizability of the models to new compounds, such models may still be worthy of investigation. The kernel-based multitask modeling approach itself should be of interest to investigators, and furthermore it is possible that the models could be of use in drug discovery. In a small application of the models, Boik et al. [1] used them to help identify several dozen compounds that were predicted to be active *in-vitro *against the three NCI cell lines, were predicted to have low rat LD50 values, and were commercially available. Of these, 22 were tested *in-vitro *and 8 were sufficiently water-soluble and cytotoxic in a specific 48-hour assay to allow their use in the study.

## Conclusion

The results shown here suggest that in some cases, multitask learning can be useful for constructing QSAR models. Depending upon the multi-task model, precision was improved over single-task models and computation time was reduced. When applied to a large natural compound library, the models developed here for cytotoxicity, LD50, and oral clearance identified an active set of about 400 compounds that was rich in flavonoids and anthraquinones. This is the first published report of an oral clearance QSAR model that used only chemical information as explanatory variables.

## Methods

### Data sets used

Rat LD50 values were taken from the Registry of Toxic Effects of Chemical Substances (RTECS) database [55] and cytotoxicity values were taken from the public National Cancer Institute (NCI) Developmental Therapeutics Program database [56]. For the LC50 and cytotoxicity data sets, compounds were excluded if they were not cyclic, were of molecular weight greater than 700 grams/mole, or were composed of any atoms other than carbon, oxygen, nitrogen, sulfur, and/or hydrogen. These criteria were intended to select compounds that resemble drug-like natural products. The cytotoxicity model consisted of submodels for three human cancer cell lines: lung NCI-H460, breast MCF7, and glioblastoma SF-268. These lines were selected because they were employed in the NCI prescreening process. Each cell line submodel itself consisted of two submodels: LC50 (concentration of drug resulting in a 50 percent reduction in the measured cellular protein content) and TGI (concentration of drug producing total growth inhibition).

The oral clearance model used bioavailability as a second, related task. Bioavailability was included only to improve the accuracy of oral clearance predictions, and so bioavailability predictions are not reported. Oral clearance and bioavailability values were taken from the literature (see Additional File 2). Wherever possible, oral clearance and bioavailability values were obtained for healthy adults. Averaged values of oral clearance or bioavailability were used if more than one value per drug was available, and compounds were excluded if the deviation of reported values was excessive. Compounds were also excluded if their molecular weight was greater than 900 grams/mole or if they were composed of atoms other than carbon, oxygen, nitrogen, sulfur, chlorine, fluorine, and hydrogen. Less restrictive exclusion criteria were used for the oral clearance and bioavailability data compared to the cytotoxicity and LD50 data in order to obtain a suitable number of records for training; relatively few published oral clearance values were available. This means that while predictions can be made for a greater variety of compounds with the oral clearance model relative to the other models, the accuracy of these predictions will be lower – a wider chemical space is being modeled with a smaller number of training records. Descriptor values used in the oral clearance models are available from the author by request.

The library of 115,000 natural compounds was constructed using structures from the CrossFire Beilstein database [57], the PhytochemicalDB [58], and Dr. Duke's Phytochemical and Ethnobotanical Databases [59]. Natural compounds were included if they were cyclic, of molecular weight less than 800 grams/mole, of natural origin, and composed only of carbon, oxygen, nitrogen, sulfur, and/or hydrogen. The selection criteria were less restrictive than that for the LD50 and cytotoxicity data (molecular weight of <800 vs. <700 grams/mole, respectively). Less than four percent of the natural compounds had a molecular weight greater than 700, and very few of these would be considered as multivariate outliers to the LD50 and cytotoxicity training sets. None of the compounds passing the most restrictive screen listed in Table 4 were of molecular weight greater than 700.

Characteristics of the data sets are summarized in Tables S.1 and S.3 of Additional File 1. The threshold values listed in the tables (83 L/hr for oral clearance, 1,920 mg/kg for LD50, 50 *μ*M for LC50, and 10 *μ*M for TGI) were used to transform continuous responses into binary +1 and -1 labels for classification, which are referred to in the text as positive and negative labels, respectively. Classification models were constructed rather than regression because higher accuracy could be obtained. As part of a multitask model for oral clearance, however, bioavailability was modeled as a regression problem. This is because higher accuracy was obtained for the oral clearance classification model when bioavailability was modeled in a regression setting. In the multitask model for oral clearance and bioavailability there were 526 records.

### Software used

Structural features were generated using five software programs: Dragon [60], Molconn-Z [61], Molecular Modeling Pro [62], Recon [63], and JChem [64]. The number of features generated by each was Dragon (929 features), Molconn-Z (1191 features), Molecular Modeling Pro (246 features), Recon (248 features), and JChem (63 features). In total, 2,671 features were generated. After duplicate and completely correlated features were removed, approximately 1,610 were available for modeling, depending on the training set. No attempt was made to presuppose the relative importance of individual features. Because the *in-vivo *three-dimensional conformation of a compound is often not known and some (low-energy) conformers can be computationally expensive to identify, features were based on two-dimensional chemical representations. Python [65] with Numpy/Scipy [66], and MATLAB [67] environments were used for modeling.

### The KMLA Algorithm

The KMLA algorithm [8,9] is used here with minor changes. A short mathematical explanation of the KMLA algorithm is given in Additional File 3 and a brief overview is presented below. The algorithm is an extension to kernel PLS, as used by Deng et al. [68].

KMLA uses a kernel function to transform the feature space into a symmetric, positive-definite similarity matrix. Learning occurs via a PLS-like algorithm on the kernel matrix (i.e., in the distance space), rather than on the original features. Denote the original feature matrix by **X **∈ *R*^*n *× *m *^and responses by **Y **∈ *R*^*n *× *k *^for *k *tasks. Let a single subscript denote a column of a matrix (e.g., **Y**_*g*_) or a single entry of a row vector. The algorithm consists of applying a kernel function to **X**, thereby creating a kernel matrix **K **∈ *R*^*n *× *n*^. Next, columns *i *= 1, 2,... *z *of a matrix of linear orthogonal latent variables, **T **∈ *R*^*n *× *z*^, are iteratively generated from **K**, with *z *≪ *n*. For example, in the oral clearance/bioavailability model, *z *= 3 and *n *= 526. The goal is to generate **T **in such a way that it is a linear projection of **K **into a reduced subspace and the loss function $L=\sum_{g=1}^{k}\sum_{i=1}^{n}\delta_{i,g}f_{YF}\left(Y_{i,g},F_{i,g}\right)$ is minimized. Here, **F **= **TC**, where **F **∈ *R*^*n*, *k*^, is a matrix of predicted values, **C **∈ *R*^*z*, *k *^is a matrix of coefficients, and *δ*_*i*, *g *_= 0 if **Y**_*i*, *g *_is missing and *δ*_*i*, *g *_= 1 otherwise. Note that a separate model is constructed for each task and each task has its own vector of coefficients. The only thing in common between tasks is that all use the same matrix of latent variables, **T**. As such, it is reasonable to allow tasks to be based on different sets of records.

The loss function can vary between tasks. For linear regression on task *g*, *f*_*YF *_= (**Y**_*i*, *g *_- **F**_*i*, *g*_)^2^. Other loss functions could be used if desired. For binary classification on task *g*, targets are labeled as +1 and -1 and an exponential loss function is used, *f*_*YF *_= *y*_*i *_exp(-**Y**_*i*, *g*_**F**_*i*, *g*_). Weights *y*_*i *_allow cost-sensitive learning and can be based on the relative frequency of the positive and negative labels. After learning in the subspace is completed, the matrix of PLS coefficients, **C**, is transformed to a matrix of kernel coefficients, **B**, such that predictions can be calculated as **F **= **μ **+ **KB**, where **μ **is a vector of coefficients for a constant hypothesis.

Note that KMLA is only one approach to kernelized PLS. Another, proposed by Rosipal [69] uses a PLS algorithm within the reproducing kernel Hilbert space (RKHS) produced by a kernel function. That is, learning is done in the RKHS rather than in the distance space.

KMLA (common feature mapping) is also only one approach to multi-task learning. Yu and Tresp [26] discuss several others, including Regularized Multi-task Learning and Parametric Bayesian Multi-task Learning. Another innovative form of multitask learning is to redesign molecular descriptors to be suitable for modeling multiple tasks as a single task [70].

### Model training

The model training and prediction process occurred in two phases, as listed in Table 5. The purpose of Phase I was to identify an optimal set of model parameters for a given task and to estimate model accuracy. The purpose of Phase II was to build predictive models using the optimal parameters identified in Phase I.

**Table 5:** Modeling phases and data partitions

**Phase**	**Partitions/activity**	**Oral Clearance**	**LD50**	**Cytotoxicity**
Phase I	training set	348 of 435 (80%)	3,095 of 3,869 (80%)	4,000 of 8,983 (44%), chosen from 70%
	testing set	87 of 435 (20%)	774 of 3,869 (20%)	2,695 of 8,983 (30%)
Phase II	training set	435 of 435 (100%)	3,869 of 3,869 (100%)	4,500 of 8,983 (50%), includes all positive labels

In Phase I, models were replicated numerous times to better evaluate accuracy. For each replicate model, the complete data set was randomly partitioned into training and testing sets. The test set was not used for any training, parameter selection, record selection, or feature selection. After partitioning, features that were of constant value or completely correlated with others in the training set were removed. Training data were further divided into 10 sets for cross-validation. For the models constructed in Phase II, all records (or a selected subset of records for the cytotoxicity model) were used in training and no test set was created. As in Phase I, training data were partitioned into 10 cross-validation sets.

Model averaging was employed in both Phase I and II to help increase the predictive power of the models. The noisy nature of the modeled responses, as well as the weak relationships between features and responses, made construction of accurate models a challenging task. Binary predictions from the 10 cross-validation models were averaged; average values less than zero were labeled -1, and those zero or above were labeled +1.

### Model selection

To use the KMLA algorithm, the number of latent features (the parameter *z *described above) must be specified. In this paper, no more than four latent features were used for any model constructed. The choice of latent features was determined from the training set results – only those latent features that greatly affected training set accuracy were retained.

The kernel type and any associated kernel parameters also must be specified. A Gaussian kernel function was employed for all nonlinear models constructed here. The Gaussian kernel has one parameter that must be chosen, kernel width (*σ*^2^). Based on results from several training sets, *σ*^2 ^= 500 was used for all Gaussian kernels. Predictions were not very sensitive to small changes in *σ*^2^.

Lastly, when used for classification the KMLA algorithm requires that a threshold parameter (the parameter *ψ *described in Additional File 3) be specified for separating the classes. This parameter is tuned to produce similar precision for positive and negative labels and is determined based on results of 10-fold cross-validation of the training records.

### Record selection

The NCI data set contained 8,983 records, which was too large to model in a reasonable length of time. In addition, the number of positive labels in the data set was much smaller than the number of negative ones (see Table S.3 of Additional File 1 for the distribution of positive labels). Therefore, all records with positive labels in the training set were retained, along with a subset of records with negative labels. The subset was constructed by two methods: random selection and an iterative method.

In the iterative method, 2,000 negative-label records were randomly chosen to form a training set (along with all positive-label records). Model parameters were estimated and scores were given to each negative-label record based on the sum of the absolute values of the vector of coefficients for that record, taken from the matrix **B **(see above). The reasoning was that if values taken from **B **were small for a given record, that record would not greatly influence predictions and it could be assumed that the record was relatively unimportant to the model. In subsequent iterations, some records were randomly selected for training and scoring, and some were selected based on their average scores from previous iterations. After all negative-label records had been selected at least twice, they were ranked by score and 4,000 records in total were selected for the Phase I training set (4,500 were selected for the Phase II training set). Note that record selection was based on the training records only – test set records were not used for any training or record selection. Therefore results from the test sets can be used to assess the two record selection procedures.

### Feature selection

To improve the accuracy of the oral clearance model, an iterative backwards elimination feature selection algorithm was used. In each iteration, features were removed that did not contribute greatly to predictions. More specifically, in each iteration a model was constructed using a data set of *m *features and *n *rows, and predictions were made for the training set. In the first iteration, *m *equaled the total number of available features. Five models were created, where the number of retained latent features in each was three to seven, inclusive. Thus, five predictions were made for each training point and predictions formed a matrix $\hat{Y}$ ∈ *R*^*n *× 5^. Next, *m *additional $\hat{Y}$ matrices were produced, each one for a data set where one of the *m *features was omitted. The score for the *i*th feature was calculated as $S_{i}=\left\|{\hat{Y}}_{m}-{\hat{Y}}_{-i}\right\|$, where the subscript *m *refers to use of all available features and the subscript -*i *refers to use of all available features except feature *i*. If removal of feature *i *did not alter the predictions at all, the score *S*_*i *_would be equal to zero. Features with a score less than 5 percent of the maximum score for that iteration were removed and a new iteration was started using the reduced feature set. No more than 15 percent of the available features were removed in any single iteration. The iterations continued until the scores for all remaining features were greater than 5 percent of the maximum score for that iteration. Roughly 80 percent of all features were retained using this algorithm.

A variety of other feature selection methods have been proposed in the literature and could have been used. For example, genetic algorithms have been used for feature selection in QSAR studies [71]. The feature selection algorithm described above was chosen because it could handle large numbers of features (including large numbers of retained features), and because it could serve as a wrapper for the KMLA algorithm.

## Authors' contributions

JCB developed the modeling approach, coded the software, and was the primary author of the manuscript. RAN reviewed the study design, participated in coordination of the study, and helped draft the manuscript.

## Supplementary Material

Additional File 1 Supplemental Tables. Three additional tables that summarize data sets and results. Click here for file

Additional File 2 Oral clearance and bioavailability values. A table of oral clearance and bioavailability values used in the manuscript. Click here for file

Additional File 3 A short mathematical explanation of the KMLA algorithm. A short mathematical explanation of the KMLA algorithm. Click here for file
