# Supplementary material for: Structure-activity models of oral clearance, cytotoxicity, and LD50: a screen for promising anticancer compounds
Source: BMC Pharmacol. 2008 Jun 13;8:12. doi: 10.1186/1471-2210-8-12 (PMC2442056; doi:10.1186/1471-2210-8-12)
Supplement: Additional File 1 — Supplemental Tables. Three additional tables that summarize data sets and results. [file 1471-2210-8-12-S1.doc]

# Additional File 1: Supplemental Tables

## Table S.1. Characteristics of the oral clearance and LD50 data

| **Characteristic** | **Oral Clearance** | | **Rat LD50** |
| --- | --- | --- | --- |
|  | **Bioavailability** | **Oral Clearance** | **LD50** |
| Number of records | 309 | 435 | 3,869 |
| Number of +1 labels | – | 305 | 1,276 |
| Fraction of +1 labels | – | 0.70 | 0.33 |
| Threshold | – | ~83 L/hr ^a^ | ~1,920 mg/kg ^b^ |
| Label for low values | – | +1 | –1 |
| a) The threshold was the 70^th^ percentile of oral clearance values in a training set.  b) The threshold was the 66^th^ percentile of LD50 values in a training set. | | | |

## Table S.2. Effects of model averaging

| **Data partition** | **Average precision, OC.1** | **Average precision, OC.3** |
| --- | --- | --- |
| Training, (+) labels | 0.757 ((0.029) | 0.748 (0.019) |
| Training, (–) labels | 0.864 (0.025) | 0.817 (0.024) |
|  |  |  |
| Test set, (+) labels,  w/ model averaging | 0.649 (0.060) | 0.636 (0.060) |
| Test set, (+) labels,  w/o model averaging | 0.623 (0.052) | 0.621 (0.058) |
|  |  |  |
| Test set, (–) labels,  w/ model averaging | 0.569 (0.111) | 0.625 (0.091) |
| Test set, (–) labels,  w/o model averaging | 0.553 (0.088) | 0.598 (0.068) |

### Table S.3. Characteristics of the cytotoxicity data

| **Characteristic** | **H460  LC50** | **H460 TGI** | **MCF7 LC50** | **MCF7 TGI** | **SF-268 LC50** | **SF-268 TGI** |
| --- | --- | --- | --- | --- | --- | --- |
| Number of records | 8,983 | 8,983 | 8,983 | 8,983 | 8,983 | 8,983 |
| Number of +1 labels | 511 | 467 | 487 | 453 | 349 | 310 |
| Fraction of +1 labels | 0.057 | 0.052 | 0.054 | 0.05 | 0.039 | 0.035 |
| Threshold | 50 μM | 10 μM | 50 μM | 10 μM | 50 μM | 10 μM |
| Label for low values | +1 | +1 | +1 | +1 | +1 | +1 |
