# Supplementary material for: Structure-activity models of oral clearance, cytotoxicity, and LD50: a screen for promising anticancer compounds
Source: BMC Pharmacol. 2008 Jun 13;8:12. doi: 10.1186/1471-2210-8-12 (PMC2442056; doi:10.1186/1471-2210-8-12)
Supplement: Additional File 2 — Oral clearance and bioavailability values. A table of oral clearance and bioavailability values used in the manuscript. [file 1471-2210-8-12-S2.pdf]

Additional file 2: Oral clearance and bioavailability values

The oral clearance and bioavailability data modeled are listed in the table below.

Drug	Bio-availability	Bioavailability References	Oral Clearance (L/hr)	Oral Clearance References
11-keto beta-boswellic acid	–	–	20.5	[1]
abacavir	–	–	58.89	[2, 3]
acetaminophen	0.78	[2, 4, 5]	17.46	[4, 6]
acetanilide	–	–	32.5	[7]
acetazolamide	–	–	2.68	[8]
acetyl-11-keto beta-boswellic acid	–	–	63.8	[1]
acrivastine	–	–	17.15	[4, 9]
actisomide	–	–	70.95	[10]
acyclovir	0.20	[4, 5, 11]	82.32	[4, 12]
adinazolam	0.39	[13]	–	–
albendazole	–	–	86.52	[2]
alfentanil	–	–	88	[14]
alfuzosin	–	–	52	[15]
allopurinol	0.59	[2, 4, 5]	59.07	[4, 16, 17]
almotriptan	–	–	52.2	[18]
alosetron	0.55	[19, 20]	62	[20]
alpha-boswellic acid	–	–	16	[1]
alprazolam	0.88	[2]	–	–
amantadine	0.85	[4, 5]	28.93	[4]
amiloride	0.5	[5]	–	–
amineptine	–	–	125	[21]
aminoglutethimide	0.9	[5]	7.87	[22]
aminopyrine	–	–	12.39	[23]
amisulpride	0.46	[24]	77.45	[25, 26]
amitriptyline	0.48	[4, 5]	96	[4]
amlodipine	0.68	[2, 5]	46.68	[27]
amosulalol	–	–	10.12	[10]
amoxicillin	0.93	[2, 5]	12.01	[10]
amphetamine	–	–	7.56	[2]
ampicillin	0.5	[5]	30.5	[28]
anastrozole	0.8	[2]	2.3	[29]
antipyrine	–	–	2.55	[23, 30]

Drug	Bio-availability	Bioavailability References	Oral Clearance (L/hr)	Oral Clearance References
apomorphine	–	–	280	[31]
aprepitant	0.625	[2]	–	–
aripiprazole	0.87	[2, 19]	3.49	[2]
arteflene	–	–	617.4	[10]
artemisinin	–	–	261	[32]
ascorbic acid	–	–	4.2	[33, 34]
aspirin	0.68	[2, 5]	–	–
atazanavir	–	–	17.99	[2, 35]
atenolol	0.54	[2, 4, 5]	16.09	[4, 36]
atomoxetine	–	–	27.89	[2, 37]
atorvastatin	0.13	[2, 5]	121.8	[2]
atropine	0.5	[5]	–	–
AY-30-068	–	–	14.7	[10]
azathioprine	0.8	[5]	–	–
azithromycin	–	–	125.9	[2, 10]
azosemide	–	–	2725	[38]
baclofen	0.7	[5]	11.42	[2]
balofloxacin	–	–	12.52	[10]
bambuterol	0.09	[39]	899	[39, 40]
barnidipine	–	–	3046	[10, 41]
benazeprilat	–	–	7.3	[42]
bendroflumethiazide	0.9	[5]	–	–
bepridil	0.65	[4]	49.7	[4, 27]
beta-boswellic acid	–	–	13.65	[1, 43]
betamethasone	0.8	[4]	–	–
betaxolol	0.9	[4]	21.21	[4, 44-46]
bicalutamide	–	–	15.54	[2]
biperiden	0.33	[47]	–	–
bisoprolol	0.9	[4]	17.22	[4]
bosentan	0.47	[11, 48]	21.7	[48]
budipine	–	–	10.5	[49]
bumetanide	0.9	[5]	13.87	[2, 50]
buprenorphine	–	–	461	[51]
bupropion	–	–	151.2	[2]
buspirone	0.039	[2]	3161	[2, 52]
busulfan	0.7	[2]	18.9	[2]
cabergoline	–	–	295	[53]
caffeine	0.95	[4]	6.21	[4]
calcitriol	–	–	2.96	[2]
candoxatril	–	–	24.96	[27]
captopril	0.69	[4, 5]	81	[42]

Drug	Bio-availability	Bioavailability References	Oral Clearance (L/hr)	Oral Clearance References
carbamazepine	0.72	[5]	–	–
carteolol	0.85	[4]	41.18	[4]
carvedilol	0.28	[2, 19]	146.2	[2]
cefaclor	–	–	32.68	[10]
cefadroxil	–	–	9.165	[54, 55]
cefazolin	0.95	[2]	4.2	[2]
cefdinir	0.20	[2, 56]	47.05	[2, 10, 56]
cefditoren pivoxil	0.14	[19]	–	–
cefixime	0.47	[2]	14.79	[1, 2, 10, 43, 57]
centchroman	–	–	6.35	[58]
cephalexin	0.9	[2, 5]	19.5	[10] [2]
cetirizine	–	–	3.11	[2]
chloramphenicol	0.83	[5]	–	–
chlordiazepoxide	1	[5]	–	–
chloroquine	0.8	[2]	–	–
chlorothiazide	0.2	[5]	–	–
chlorpheniramine	0.41	[2]	–	–
chlorproguanil	–	[2]	89.6	[59]
chlorpromazine	0.32	[2]	36.12	[2]
chlorthalidone	0.7	[5]	–	–
chrysin	–	–	6250	[60]
CI-1007	–	–	1818	[10, 27]
cicaprost	0.97	[61]	18.25	[61, 62]
cilazapril	–	–	13.1	[42, 63]
cilazaprilat	–	–	16	[42]
cimetidine	0.63	[2, 4, 5, 64]	57.45	[2, 4, 64]
cinacalcet	0.2	[2]	–	–
cinmetacin	–	–	4.8	[65]
ciprofloxacin	0.63	[2, 5, 11]	–	–
citalopram	0.8	[19]	25.14	[2, 19]
cladribine	0.5	[5]	–	–
clarithromycin	0.54	[2, 4]	39.46	[2, 4, 10]
clentiazem	–	–	147.8	[10]
clindamycin	0.89	[2, 5]	–	–
clofazimine	–	–	76.7	[11, 66, 67]
clonazepam	0.94	[2, 5, 11]	6.51	[2]
clonidine	0.89	[2, 5]	–	–
cloxacillin	0.45	[5]	–	–
clozapine	0.55	[2]	–	–
codeine	0.5	[4]	59.635	[2, 4, 68]

Drug	Bio-availability	Bioavailability References	Oral Clearance (L/hr)	Oral Clearance References
colchicine	–	–	36.61	[69, 70]
coumarin	0.034	[71]	2069	[71]
cycloserine	–	–	2.4	[72]
cytarabine	0.2	[4, 5]	275	[4]
dapsone	0.93	[2]	–	–
darifenacin	–	–	205.8	[10]
delapril	–	–	52	[42]
delavirdine	–	–	58.92	[2, 73]
desipramine	0.4	[4]	101.3	[4]
desloratadine	–	–	72.1	[74]
dextromoramide	–	–	14.7	[75]
diazepam	0.97	[2, 5]	2.94	[10]
diclofenac	0.55	[2, 19]	34.9	[19]
dicloxacillin	0.62	[2, 5]	–	–
didanosine	0.4	[4, 5]	160.1	[2, 4, 76]
dienogest	–	–	6.174	[10]
diethylcarbamazine	–	–	20.1	[77, 78]
digitoxin	–	–	0.26	[79]
digoxin	0.7	[2]	17.77	[4, 80]
dihydrocodeine	–	–	39.5	[81]
diltiazem	0.41	[2, 4, 11]	161	[2, 4, 10, 27]
diphenhydramine	0.64	[2, 4]	52.17	[2, 4]
dipyrone	–	–	156	[82]
disopyramide	0.83	[5]	–	–
dofetilide	0.96	[2, 19]	21.76	[10]
dolasetron	0.9	[5]	–	–
domperidone	0.16	[5]	–	–
donepezil	1	[83]	11.42	[2, 10, 84]
dosulepin	–	–	95.2	[85]
doxapram	0.6	[5]	–	–
doxazosin	0.66	[4]	9.03	[4, 10]
doxepin	0.3	[4, 5]	148.5	[4, 86]
doxorubicin	0.05	[2, 5]	–	–
doxycycline	0.94	[2, 4, 5]	2.32	[4]
doxylamine	–	–	12	[87]
droloxifene	–	–	34.8	[88]
dronabinol	0.15	[4]	106	[4]
duloxetine	–	–	61.6	[89]
dyphylline	–	–	12.3	[90]
efavirenz	–	–	12.69	[2, 91]
EGCG	–	–	928	[92]

Drug	Bio-availability	Bioavailability References	Oral Clearance (L/hr)	Oral Clearance References
emtricitabine	–	–	5.28	[93]
enalapril	0.6	[5]	20.58	[2]
enoxacin	–	–	22.89	[10, 27]
enoximone	–	–	99	[94]
entacapone	0.39	[2, 19]	–	–
epanolol	–	–	2535	[95, 96]
ephedrine	1	[2]	28.86	[2, 4]
epinastine	–	–	70.9	[96]
eprosartan	0.13	[19]	87.1	[97]
ergometrine	–	–	48	[98]
ergotamine	–	–	32790	[99]
ethambutol	0.77	[2, 5]	–	–
ethinyl estradiol	0.44	[4, 5, 11]	43.35	[4, 100]
ethosuximide	–	–	0.77	[4, 10]
etodolac	–	–	3.43	[11]
etoposide	0.51	[2, 4, 5]	5.36	[4]
etoricoxib	–	–	4.1	[101]
exemestane	–	–	609	[2]
ezetimibe	–	–	27.72	[2]
famotidine	0.43	[4, 5, 11, 102]	68.29	[4]
felbamate	–	–	2.52	[2, 10]
felodipine	0.18	[2, 5]	291.5	[10]
fenofibrate	0	[19]	1.89	[2]
fenoterol	0.02	[103]	–	–
feprazone	–	–	0.84	[104]
fexofenadine	0.32	[5, 105]	45.37	[2, 105, 106]
finasteride	0.72	[2, 5]	–	–
FK-1052	–	–	110.9	[10]
flecainide	0.79	[2, 5]	–	–
fleroxacin	–	–	14.34	[27]
fluconazole	0.93	[2, 5]	1.68	[10]
flucytosine	0.82	[5]	–	–
flunitrazepam	0.9	[5]	–	–
flunoxaprofen	–	–	1.59	[10]
fluorouracil	0.3	[5]	–	–
fluoxetine	–	–	40.32	[2]
fluphenazine	0.027	[2]	–	–
flutamide	–	–	1176	[2]
fluvastatin	0.24	[5]	–	–
fradafiban	–	–	24.6	[27]
frovatriptan	0.25	[19]	46.6	[107]

Drug	Bio-availability	Bioavailability References	Oral Clearance (L/hr)	Oral Clearance References
furosemide	0.68	[2, 5]	–	–
fusidic acid	0.91	[108]	1.36	[108]
gabapentin	0.6	[2, 4, 5]	11.26	[4, 11, 109, 110]
galantamine	0.97	[2, 11, 83]	19.7	[111, 112]
gallopamil	–	–	330	[113, 114]
ganaxolone	–	–	747.6	[10]
ganciclovir	0.039	[2, 4]	351.6	[2, 4, 115]
gefitinib	0.6	[2]	84.5	[116]
gemfibrozil	0.95	[2, 4, 5]	7.30	[4]
gestodene	–	–	241	[117, 118]
GI-147211	–	–	316	[119]
glimepiride	0.98	[2, 4]	3.16	[4]
glipizide	0.93	[2, 4, 5]	2.24	[2, 4, 120]
glyburide	0.8	[5]	–	–
glycopyrrolate	–	–	769	[121]
granisetron	0.6	[2]	77	[2]
grepafloxacin	–	–	24.78	[10]
haloperidol	0.65	[2, 5]	–	–
hexobarbital	–	–	15.1	[27, 122]
HI-6	–	–	402	[123]
hydralazine	0.4	[5]	–	–
hydrochlorothiazide	0.71	[2, 5]	–	–
hydrocodone	–	–	37.5	[4]
hydrocortisone	–	–	17.7	[124]
hydromorphone	0.42	[2]	146	[2]
hydroxyurea	1	[2]	–	–
hydroxyzine	–	–	41.16	[2]
hyperforin	–	–	13.4	[125]
hypericin	–	–	6.1	[125, 126]
ibuprofen	0.93	[5]	4.8	[2, 4, 127]
idarubicin	0.27	[2, 5]	417.5	[2, 128]
idazoxan	0.34	[129]	–	–
ifetroban	–	–	54.6	[10]
imatinib	0.98	[2, 130]	–	[130-132]
imidapril	–	–	88.2	[10]
imipramine	0.46	[2, 4, 5, 133]	168.8	[4, 134]
inamrinone (amrinone)	–	–	16	[135]
indapamide	0.9	[5]	–	–
indinavir	–	–	79.5	[27]
indomethacin	0.99	[2, 5]	–	–

Drug	Bio-availability	Bioavailability References	Oral Clearance (L/hr)	Oral Clearance References
irbesartan	0.7	[2, 5, 11, 136]	12.72	[2]
isoniazid	–	–	23.31	[2]
isosorbide dinitrate	0.22	[2, 5, 137]	878.2	[2]
isosorbide mononitrate	0.92	[2, 5]	8.13	[2]
isoxicam	1	[138]	0.51	[139]
isradipine	0.2	[4]	244	[2, 4, 140]
itraconazole	0.55	[2]	96.6	[2]
ivermectin	–	–	8.65	[2]
KC-764	–	–	18.73	[10]
ketamine	0.2	[5]	–	–
ketanserin	–	–	54.6	[10]
ketoconazole	–	–	35.28	[2]
ketoprofen	0.98	[4]	6.8	[4, 141]
ketorolac	0.95	[2, 4]	2.22	[4]
ketotifen	–	–	0.86	[142]
KG-2413	–	–	73.5	[10]
labetalol	0.23	[4, 143]	333.3	[4, 144, 145]
lacidipine	–	–	487.2	[10]
lamivudine	0.83	[2, 5, 11]	–	–
lamotrigine	0.96	[2, 5]	2.06	[2]
lansoprazole	0.83	[2, 5]	–	–
leflunomide	–	–	0.034	[2, 146]
lercanidipine	–	–	1312	[147]
letrozole	1.0	[2]	1.52	[148]
leucovorin	–	–	6.9	[149]
levetiracetam	1	[2]	–	–
levodopa	0.43	[2, 5]	–	–
levonorgestrel	1	[19]	6.53	[4, 150]
levosimendan	0.85	[151]	25.3	[151]
lidocaine	0.35	[2, 4, 5]	112.3	[2, 4]
limonene	–	–	49.7	[152]
linezolid	1	[2]	–	–
lipoic acid	–	–	184	[153-155]
lisinopril	0.33	[2, 5]	17.64	[2]
lisuride	–	–	320	[53]
lomefloxacin	–	–	15.5	[10]
loperamide	0.4	[5]	–	–
lopinavir	–	–	4.57	[2, 156]
loratadine	–	–	774	[157]
lorazepam	0.94	[2, 5]	–	–
losartan	0.34	[2, 5]	–	–

Drug	Bio-availability	Bioavailability References	Oral Clearance (L/hr)	Oral Clearance References
lutein	–	–	10.8	[158]
malotilate	–	–	7080	[159]
mebendazole	0.22	[160]	289.9	[160]
medroxyprogesterone	–	–	642.6	[2]
mefloquine	–	–	1.81	[2]
meloxicam	0.93	[2, 19]	0.45	[10, 161]
melphalan	0.71	[2]	–	–
meptazinol	–	–	1801	[2, 162-164]
mercaptopurine	0.20	[2, 5, 165]	385	[2]
metaxalone	–	–	58.8	[2]
metformin	0.54	[2, 5]	–	–
methadone	0.89	[2, 4]	8.63	[2, 4]
methazolamide	–	–	0.037	[166]
methotrexate	0.69	[2, 5]	10.5	[167, 168]
methotrimeprazine (levomepromazine)	–	–	169.8	[169]
methyldopa	0.5	[5, 170]	82.8	[170]
methylprednisolone	0.82	[2, 4, 5]	30.32	[4, 171]
metoclopramide	0.68	[2, 5]	–	–
metoprolol	0.42	[2, 4, 5]	134.8	[2, 4, 172]
metronidazole	0.95	[2, 5]	–	–
mexiletine	0.9	[5]	37.4	[173-175]
mianserin	0.3	[5]	–	–
midazolam	0.44	[2]	243	[176]
mifentidine	–	–	39	[177]
mifepristone (RU486)	–	–	1.04	[178]
minaprine	–	–	12.06	[27]
minocycline	0.99	[4, 5]	3.84	[2, 4, 179]
minoxidil	–	–	99	[180]
mirtazapine	0.5	[2]	58.15	[2, 181]
modafinil	–	–	3.7	[182]
modipafant	–	–	70.56	[10]
mofarotene	–	–	31.5	[10]
molsidomine	–	–	55.2	[183]
montelukast	0.675	[2, 11, 184]	3.42	[11, 184]
morphine	0.30	[2, 5, 11]	–	–
morphine-6-glucuronide	0.11	[185]	94.3	[185]
moxifloxacin	0.88	[2, 19]	11.82	[27]
mycophenolate mofetil	–	–	15.6	[186]
mycophenolic acid	–	–	17.2	[187]

Drug	Bio-availability	Bioavailability References	Oral Clearance (L/hr)	Oral Clearance References
nadolol	0.35	[4]	35.71	[4]
nafcillin	0.36	[4]	83.33	[4]
nalbuphine	–	–	832.5	[2, 10, 188]
nalmefene	0.4	[2]	157.5	[2]
naloxone	0.033	[2, 4, 5]	4560	[2, 4]
naltrexone	0.2	[5]	–	–
naproxen	0.99	[2]	0.56	[2, 4, 189]
naratriptan	–	–	35.45	[2, 190]
nefopam	–	–	209	[191]
netivudine	–	–	4.62	[192]
nevirapine	0.93	[2]	2.4595	[2, 193]
nicardipine	–	–	549	[10, 27]
nicotine	0.3	[4]	325.8	[2, 4, 194]
nifedipine	0.55	[2, 4, 5]	60.97	[4, 195, 196]
nilutamide	–	–	4.0	[10]
nilvadipine	–	–	432.6	[10, 27]
nimesulide	–	–	5.5	[197]
nimodipine	0.09	[5, 198]	1216	[198]
nisoldipine	–	–	1102	[10, 199]
nitrazepam	0.8	[5]	4	[200]
nitrendipine	0.11	[4]	1233	[4, 201]
nitrofurantoin	0.87	[2]	47.79	[2]
nizatidine	0.9225	[4, 5, 202]	46.77	[4, 10, 202, 203]
norethindrone	0.65	[4]	38.46	[4]
norfloxacin	0.45	[5]	92.82	[10]
nortriptyline	0.51	[2, 4, 5]	60.4	[2, 4]
O6-benzylguanine	–	–	53.2	[204]
ofloxacin	0.99	[19]	9.53	[2, 10]
olanzapine	–	–	23.31	[2, 205, 206]
omeprazole	0.45	[4, 5]	69.64	[2, 4, 10, 207]
ondansetron	0.59	[4, 5]	52.47	[2, 4, 208]
orciprenaline	0.4	[5]	–	–
oseltamivir	–	–	630	[2]
oxacillin	0.33	[4]	77.27	[4]
oxaprozin	–	–	0.29	[209]
oxipurinol	–	–	1.5	[17]
oxprenolol	0.5	[5]	55.3	[210]
oxybutynin	0.063	[2]	544.3	[2]
oxycodone	0.50	[5, 211, 212]	53.6	[211]
pantoprazole	0.83	[5, 11]	–	–

Drug	Bio-availability	Bioavailability References	Oral Clearance (L/hr)	Oral Clearance References
paroxetine	–	–	36.12	[2]
pazufloxacin	–	–	26.42	[10]
pefloxacin	–	–	8.28	[27]
pelrinone	–	–	31.71	[10]
penbutolol	0.85	[4]	23.53	[4]
pentazocine	–	–	450	[213]
pentobarbital	0.94	[214]	2.06	[214]
pentopril	–	–	59.65	[42, 215]
pentoxifylline	0.35	[4]	745.6	[4, 216]
perindopril	0.75	[19]	17	[42]
perindoprilat	–	–	67	[42]
pethidine (Meperidine)	0.54	[2, 4, 5]	138.7	[2, 4]
phenacetin	–	–	203	[217]
phenobarbital	0.98	[2, 4]	0.27	[2, 4]
phenprocoumon	–	–	0.05	[218]
phenylbutazone	0.01	[219]	0.09	[219]
phenylephrine	0.38	[4]	305.2	[2, 4]
phenylpropanolamine	–	–	42.5	[4]
phenytoin	0.94	[2, 5]	–	–
physostigmine	0.023	[83, 220]	10690	[220]
pidotimod	–	–	10.67	[10]
pinacidil	–	–	52.08	[10]
pindolol	0.83	[4, 5]	39.23	[4, 221]
pioglitazone	–	–	5.521	[2, 11]
piperacillin	0	[19]	–	–
piritrexim	–	–	12.4	[222]
piroxicam	–	–	0.15	[4]
PNU-96391A	–	–	53.4	[223]
posaconazole	–	–	465	[224]
pramipexole	0.95	[2, 19]	32.3	[2, 53, 225]
pranlukast	–	–	90.8	[226]
pravastatin	0.22	[2, 5]	–	–
prazosin	0.69	[2, 4, 5]	19.56	[2, 4, 227, 228]
prednisolone	0.82	[2]	14	[229]
prednisone	0.8	[2]	–	–
pregabalin	0.95	[19]	–	–
prenalterol	0.25	[230]	–	–
primaquine	–	–	32.5	[4]
primidone	0.85	[5, 231]	2.01	[231, 232]
procainamide	0.85	[2, 4, 5]	31.67	[4]
prochlorperazine	0.2	[5]	–	–

Drug	Bio-availability	Bioavailability References	Oral Clearance (L/hr)	Oral Clearance References
promazine	–	–	401	[233]
promethazine	0.25	[4, 5]	260	[4]
propafenone	0.28	[4]	235.1	[4, 234]
propiram	–	–	22.4	[235]
propoxyphene	–	–	45	[4]
propranolol	0.31	[2, 5]	181	[236]
propylthiouracil	0.85	[5]	13.8	[237]
pseudohypericin	–	–	12.15	[125, 126]
pyrazinamide	–	–	4.62	[2]
quetiapine	0.09	[2]	81.15	[238, 239]
quinapril	0.55	[5]	–	–
quinine	0.77	[2, 4, 5]	19.66	[2, 240]
raloxifene	0.02	[2, 19]	3154.3	[2, 241]
ramipril	0.37	[2, 5, 11]	96.6	[2]
ramiprilat	–	–	46	[42]
ranitidine	0.59	[2, 4, 5]	65.39	[2, 4, 10]
ranolazine	–	–	142	[242]
reboxetine	–	–	1.74	[243]
recainam	–	–	44.94	[10]
remikiren	–	–	15820	[10]
repaglinide	0.56	[2]	62.88	[2, 244]
ribavirin	0.49	[2, 5]	35.22	[2, 245, 246]
rifampin	–	–	15.58	[10]
riluzole	0.6	[19]	–	–
ritonavir	–	–	4.9	[2, 19]
rivastigmine	0.36	[19]	–	–
rizatriptan	0.46	[2, 19]	145.1	[2, 247, 248]
rofecoxib	–	–	6.3	[2, 10]
rolipram	–	–	11.5	[249]
ropinirole	0.55	[2]	58.78	[2, 53]
rosiglitazone	0.99	[2]	2.86	[2]
rosuvastatin	0.2	[2]	–	–
saccharin	0.85	[250]	18.04	[250]
salicylic acid	1	[5]	–	–
scopolamine	0.27	[251]	590	[252]
selegiline	0.2	[5]	5387	[2, 253, 254]
sertraline	–	–	165.5	[2, 10]
sildenafil	0.38	[2]	68.73	[10, 27]
simvastatin	0.038	[5, 11]	–	–
sinitrodil	–	–	504	[10]
sotalol	0.95	[5]	17.95	[4, 255]

Drug	Bio-availability	Bioavailability References	Oral Clearance (L/hr)	Oral Clearance References
sparfloxacin	0.92	[11, 256]	11	[10]
spironolactone	0.8	[5]	390.6	[2]
stavudine	0.85	[4]	39.49	[4, 257]
sulfadiazine	1	[4]	2.2	[4]
sulfamethoxazole	0.95	[2, 4, 5]	1.5	[4]
sulfisoxazole	0.98	[5, 258]	1.618	[2, 258, 259]
sumatriptan	0.16	[2, 5]	75.6	[10]
SY-5555	–	–	39.94	[10]
tacrine	0.26	[5, 83]	1404	[260]
tadalafil	–	–	2.48	[2, 261]
tamoxifen	–	–	5.88	[2]
tamsulosin	1	[2]	2.167	[10]
tegaserod	0.11	[2, 19]	–	–
telithromycin	0.57	[11, 262]	100.7	[262]
temazepam	0.9	[5]	–	–
temozolomide	–	–	13.9	[263-265]
tenoxicam	1	[266]	0.11	[267]
terazosin	0.82	[2]	4.75	[268]
terbinafine	–	–	65	[4]
terbutaline	0.13	[5, 269]	100.3	[269]
terfenadine	–	–	4420	[270]
terodiline	0.9	[271]	5.55	[271]
testosterone	0.043	[5, 272]	2137	[272]
tetracycline	0.77	[2, 5]	9.11	[2]
thalidomide	–	–	9.82	[4, 273]
theophylline	0.94	[2, 4, 5]	2.84	[4]
thioridazine	–	–	55	[4]
tiagabine	0.9	[2]	9.05	[2, 274]
tiaprofenic acid	–	–	27	[275]
timolol	0.67	[2, 4, 5]	49.17	[2, 4]
tinidazole	0.95	[4]	2.21	[4]
tocainide	0.9	[4]	12.5	[4]
tolbutamide	0.89	[2, 4, 5]	0.92	[2, 4, 276]
tolcapone	0.65	[19]	10.5	[2]
tolmesoxide	0.85	[277]	15.87	[277]
toloxatone	0.56	[278]	83	[278]
tolterodine	–	–	6.8	[279]
topiramate	–	–	1.611	[2, 280, 281]
topotecan	–	–	143.1	[2, 282, 283]
torsemide	0.86	[4]	2.90	[4, 284, 285]
tosufloxacin	–	–	40.32	[10]

Drug	Bio-availability	Bioavailability References	Oral Clearance (L/hr)	Oral Clearance References
tramadol	0.76	[2, 4, 11]	31.25	[4]
tranexamic acid	0.34	[286]	20	[286]
trapidil	1	[287]	13.9	[287, 288]
trazodone	0.81	[2]	–	–
tretinoin	0.4	[2]	54.7	[2, 289]
triflusal	–	–	52.5	[10]
trimethoprim	0.95	[4, 5]	9.47	[4]
trimetrexate	0.45	[4]	6.4	[4]
trimipramine	0.4	[5]	–	–
triprolidine	–	–	60	[4]
troglitazone	–	–	43.99	[10, 27, 290]
tropisetron	0.6	[5, 291]	152	[291]
trospium chloride	0.096	[19]	11.3	[292]
trovafloxacin	0.88	[11, 293]	10.25	[10]
UK-343-664	–	–	343.6	[10]
urapidil	–	–	15.4	[294]
ursodiol (Ursodeoxycholic acid)	–	–	73.8	[295]
valdecoxib	0.83	[11, 296]	5.90	[11, 296]
valproic acid	1	[5, 11]	–	–
valsartan	0.24	[2, 19]	9.5	[297]
vardenafil	0.15	[298]	277	[298]
venlafaxine	0.28	[2]	99.4	[2, 4, 10]
verapamil	0.25	[2, 5]	–	–
viloxazine	0.85	[299]	8.89	[299, 300]
vinorelbine	0.27	[2]	252.5	[301, 302]
viqualine	0.73	[303]	120	[303]
voriconazole	0.96	[2]	–	–
warfarin	0.95	[2, 4, 5, 11]	0.18	[4, 304, 305]
xamoterol	–	–	248.5	[306, 307]
ximoprofen	0.98	[308]	7.76	[308, 309]
YM-796	–	–	46.62	[10]
yohimbine	0.22	[310]	143	[310]
zafirlukast	–	–	24.28	[2, 311]
zalcitabine	0.85	[4, 5]	19.18	[2, 4, 246, 312, 313]
zaleplon	0.31	[2]	212.7	[2]
zamifenacin	–	–	2.52	[10]
zanamivir	0.02	[314]	–	–
zidovudine	0.63	[2, 4, 5, 11]	162.8	[3, 4, 313, 315]
zileuton	–	–	39.6	[80, 316]

Drug	Bio-availability	Bioavailability References	Oral Clearance (L/hr)	Oral Clearance References
ziprasidone	0.59	[2]	–	–
zolmitriptan	–	–	120.6	[2]
zolpidem	0.71	[2, 4]	20.36	[2, 4, 317]
zonisamide	–	–	1.02	[10, 318]

A small number of errors were discovered in the bioavailability and oral clearance data sets after modeling was completed. The following incorrect bioavailability value was used in modeling: misoprostol, 0.6. The following incorrect oral clearance values were used in modeling: misoprostol, 2 L/hr; piroximone, 90.5 L/hr; prenalterol, 348 L/hr; telmisartan, 64.6 L/hr.

References

1. Sterk, V., B. Buchele, T. Simmet: **Effect of food intake on the bioavailability of boswellic acids from a herbal preparation in healthy volunteers**. *Planta Med,* 2004. **70**(12):1155-60.
2. Brunton, L., J. Lazo, K. Parker, *Goodman & Gilman's the pharmacological basis of therapeutics*. 11th ed. 2006, New York: McGraw-Hill, Medical Pub. Division.
3. Wang, L.H., G.E. Chittick, J.A. McDowell: **Single-dose pharmacokinetics and safety of abacavir (1592U89), zidovudine, and lamivudine administered alone and in combination in adults with human immunodeficiency virus infection**. *Antimicrob Agents Chemother*, 1999. **43**(7):1708-15.
4. Bertz, R.J., G.R. Granneman: **Use of in vitro and in vivo data to estimate the likelihood of metabolic pharmacokinetic interactions**. *Clin Pharmacokinet*, 1997. **32**(3):210-58.
5. Turner, J.V., D.J. Maddalena, S. Agatonovic-Kustrin: **Bioavailability prediction based on molecular structure for a diverse series of drugs**. *Pharm Res*, 2004. **21**(1):68-82.
6. Yin, O.Q., B. Tomlinson, A.H. Chow, M.S. Chow: **Pharmacokinetics of acetaminophen in Hong Kong Chinese subjects**. *Int J Pharm*, 2001. **222**(2):305-8.
7. Wynne, H.A., L.H. Cope, O.F. James, M.D. Rawlins, K.W. Woodhouse: **The effect of age and frailty upon acetanilide clearance in man**. *Age Ageing*, 1989. **18**(6):415-8.
8. Yakatan, G.J., E.L. Frome, R.G. Leonard, A.C. Shah, J.T. Doluisio: **Bioavailability of acetazolamide tablets**. *J Pharm Sci*, 1978. **67**(2):252-6.
9. Balasubramanian, R., K.B. Klein, A.W. Pittman, S.H. Liao, J.W. Findlay, M.F. Frosolono: **Pharmacokinetics of acrivastine after oral and colonic administration**. *J Clin Pharmacol*, 1989. **29**(5):444-7.
10. Wajima, T., K. Fukumura, Y. Yano, T. Oguma: **Prediction of human pharmacokinetics from animal data and molecular structural parameters using multivariate regression analysis: oral clearance**. *J Pharm Sci*, 2003. **92**(12):2427-40.
11. WebMD, *RxList, the internet drug index for prescription drug information, interactions, and side effects*. 2006, http://www.rxlist.com.

12. Steingrimsdottir, H., A. Gruber, C. Palm, G. Grimfors, M. Kalin, S. Eksborg: **Bioavailability of aciclovir after oral administration of aciclovir and its prodrug valaciclovir to patients with leukopenia after chemotherapy**. *Antimicrob Agents Chemother*, 2000. **44**(1):207-9.
13. Fleishaker, J.C., H. Friedman, S.R. Pollock: **Extent and variability of the first-pass elimination of adinazolam mesylate in healthy male volunteers**. *Pharm Res*, 1991. **8**(2):162-7.
14. Kharasch, E.D., C. Hoffer, A. Walker, P. Sheffels: **Disposition and miotic effects of oral alfentanil: a potential noninvasive probe for first-pass cytochrome P4503A activity**. *Clin Pharmacol Ther*, 2003. **73**(3):199-208.
15. Salva, P., G. Bianchetti, P. Morselli, G. Garcia-Teresa, J. Costa: **Pharmacokinetics of alfuzosin after single oral administration to healthy volunteers, of three different doses**. *Biopharm Drug Dispos*, 1992. **13**(8):583-90.
16. Murrell, G.A., W.G. Rapeport: **Clinical pharmacokinetics of allopurinol**. *Clin Pharmacokinet*, 1986. **11**(5):343-53.
17. Turnheim, K., P. Krivanek, R. Oberbauer: **Pharmacokinetics and pharmacodynamics of allopurinol in elderly and young subjects**. *Br J Clin Pharmacol*, 1999. **48**(4):501-9.
18. McEnroe, J.D., J.C. Fleishaker: **Clinical pharmacokinetics of almotriptan, a serotonin 5-HT(1B/1D) receptor agonist for the treatment of migraine**. *Clin Pharmacokinet*, 2005. **44**(3):237-46.
19. Rice, L.B.: **Antimicrobial resistance in gram-positive bacteria**. *Am J Med*, 2006. **119**(6 Suppl 1):S11-9; discussion S62-70
20. Koch, K.M., J.L. Palmer, N. Noordin, J.J. Tomlinson, C. Baidoo: **Sex and age differences in the pharmacokinetics of alosetron**. *Br J Clin Pharmacol*, 2002. **53**(3):238-42.
21. Lachatre, G., C. Piva, C. Riche, D. Dumont, R. Defrance, E. Mocaer, G. Nicot: **Single-dose pharmacokinetics of amineptine and of its main metabolite in healthy young adults**. *Fundam Clin Pharmacol*, 1989. **3**(1):19-26.
22. Alshowaier, I.A., A. el-Yazigi, A. Ezzat, A. Abd el-Warith, P.J. Nicholls: **Pharmacokinetics of S- and R-enantiomers of aminoglutethimide following oral administration of racemic drug in breast cancer patients**. *J Clin Pharmacol*, 1999. **39**(11):1136-42.
23. Kawasaki, S., H. Imamura, N. Kokudo, Y. Bandai, K. Sanjo, Y. Idezuki: **A comparison between antipyrine and aminopyrine blood clearances**. *Hepatogastroenterology*, 1992. **39**(4):344-6.
24. Rosenzweig, P., M. Canal, A. Patat, L. Bergougnan, I. Zieleniuk, G. Bianchetti: **A review of the pharmacokinetics, tolerability and pharmacodynamics of amisulpride in healthy volunteers**. *Hum Psychopharmacol*, 2002. **17**(1):1-13.
25. Coukell, A., C. Spencer, P. Benfield: **Amisulpride: A review of its pharmacodynamic and pharmacokinetic properties and theraputic efficacy in the management of schizophrenia**. *CNS Drugs*, 1996. **6**(3):237-56.
26. Hamon-Vilcot, B., S. Chaufour, C. Deschamps, M. Canal, I. Zieleniuk, P. Ahtoy, P. Chretien, P. Rosenzweig, A. Nasr, F. Piette: **Safety and pharmacokinetics of a single oral dose of amisulpride in healthy elderly volunteers**. *Eur J Clin Pharmacol*, 1998. **54**(5):405-9.

27. Mahmood, I.: **Interspecies scaling: predicting oral clearance in humans**. *Am J Ther*, 2002. **9**(1):35-42.
28. Triggs, E.J., J.M. Johnson, B. Learoyd: **Absorption and disposition of ampicillin in the elderly**. *Eur J Clin Pharmacol*, 1980. **18**(2):195-8.
29. Duan, G., J. Liang, M. Zuo: **Rapid determination of anastrozole in plasma by gas chromatography with electron capture detection and its application to an oral pharmacokinetic study in healthy volunteers**. *Biomed Chromatogr*, 2002. **16**(6):400-3.
30. Greenblatt, D.J., M.K. Divoll, J.S. Harmatz, R.I. Shader: **Antipyrine absorption and disposition in the elderly**. *Pharmacology*, 1988. **36**(2):125-33.
31. Neef, C., T. van Laar: **Pharmacokinetic-pharmacodynamic relationships of apomorphine in patients with Parkinson's disease**. *Clin Pharmacokinet*, 1999. **37**(3):257-71.
32. Rath, K., K. Taxis, G. Walz, C.H. Gleiter, S.M. Li, L. Heide: **Pharmacokinetic study of artemisinin after oral intake of a traditional preparation of Artemisia annua L. (annual wormwood)**. *Am J Trop Med Hyg*, 2004. **70**(2):128-32.
33. Blanchard, J.: **Effects of gender on vitamin C pharmacokinetics in man**. *J Am Coll Nutr*, 1991. **10**(5):453-9.
34. Blanchard, J., K.A. Conrad, P.J. Garry: **Effects of age and intake on vitamin C disposition in females**. *Eur J Clin Nutr*, 1990. **44**(6):447-60.
35. Goldsmith, D.R., C.M. Perry: **Atazanavir**. *Drugs*, 2003. **63**(16):1679-93; discussion 1694-5.
36. Lilja, J.J., K. Raaska, P.J. Neuvonen: **Effects of orange juice on the pharmacokinetics of atenolol**. *Eur J Clin Pharmacol*, 2005. **61**(5-6):337-40.
37. Chalon, S.A., J.P. Desager, K.A. Desante, R.F. Frye, J. Witcher, A.J. Long, J.M. Sauer, J.L. Golnez, B.P. Smith, H.R. Thomasson, Y. Horsmans: **Effect of hepatic impairment on the pharmacokinetics of atomoxetine and its metabolites**. *Clin Pharmacol Ther*, 2003. **73**(3):178-91.
38. Suh, O.K., S.H. Kim, M.G. Lee: **Pharmacokinetics and pharmacodynamics of azosemide**. *Biopharm Drug Dispos*, 2003. **24**(7):275-97.
39. Nyberg, L., J. Rosenborg, E. Weibull, S. Jonsson, B.M. Kennedy, M. Nilsson: **Pharmacokinetics of bambuterol in healthy subjects**. *Br J Clin Pharmacol*, 1998. **45**(5):471-8.
40. Rosenborg, J., P. Larsson, L. Nyberg: **Pharmacokinetics of bambuterol during oral administration of plain tablets and solution to healthy adults**. *Br J Clin Pharmacol*, 2000. **49**(3):199-206.
41. Teramura, T., T. Watanabe, S. Higuchi, K. Hashimoto: **Metabolism and pharmacokinetics of barnidipine hydrochloride, a calcium channel blocker, in man following oral administration of its sustained release formulation**. *Xenobiotica*, 1997. **27**(2):203-16.
42. Song, J.C., C.M. White: **Clinical pharmacokinetics and selective pharmacodynamics of new angiotensin converting enzyme inhibitors: an update**. *Clin Pharmacokinet*, 2002. **41**(3):207-24.
43. Sharma, S., V. Thawani, L. Hingorani, M. Shrivastava, V.R. Bhate, R. Khiyani: **Pharmacokinetic study of 11-Keto beta-Boswellic acid**. *Phytomedicine*, 2004. **11**(2-3):255-60.

44. Bianchetti, G., C. Blatrix, R. Gomeni, J.R. Kilborn, J. Larribaud, P.W. Lucker, J.J. Thebault, S. Trocherie, P.L. Morselli: **Pharmacokinetics of the new beta-adrenoceptor blocking agent betaxolol (SL 75212) in man after repeated oral administration**. *Arzneimittelforschung*, 1980. **30**(11):1912-6.
45. Ludden, T.M., D.A. Boyle, D. Gieseker, G.T. Kennedy, M.H. Crawford, L.K. Ludden, W.A. Clementi: **Absolute bioavailability and dose proportionality of betaxolol in normal healthy subjects**. *J Pharm Sci*, 1988. **77**(9):779-83.
46. Stagni, G., P.J. Davis, T.M. Ludden: **Human pharmacokinetics of betaxolol enantiomers**. *J Pharm Sci*, 1991. **80**(4):321-4.
47. Grimaldi, R., E. Perucca, G. Ruberto, C. Gelmi, F. Trimarchi, M. Hollmann, A. Crema: **Pharmacokinetic and pharmacodynamic studies following the intravenous and oral administration of the antiparkinsonian drug biperiden to normal subjects**. *Eur J Clin Pharmacol*, 1986. **29**(6):735-7.
48. Weber, C., R. Schmitt, H. Birnboeck, G. Hopfgartner, H. Eggers, J. Meyer, S. van Marle, H.W. Viischer, J.H. Jonkman: **Multiple-dose pharmacokinetics, safety, and tolerability of bosentan, an endothelin receptor antagonist, in healthy male volunteers**. *J Clin Pharmacol*, 1999. **39**(7):703-14.
49. Bethke, T.H., M. Merz, K. Zech, M. Seiberling, D. Hauschke, H. Heinze, W. Wurst: **Dose linearity and steady state pharmacokinetics of the new antiparkinson agent budipine after oral administration**. *Int J Clin Pharmacol Ther*, 2001. **39**(6):259-64.
50. Ward, A., R.C. Heel: **Bumetanide. A review of its pharmacodynamic and pharmacokinetic properties and therapeutic use**. *Drugs*, 1984. **28**(5):426-64.
51. Elkader, A., B. Sproule: **Buprenorphine: clinical pharmacokinetics in the treatment of opioid dependence**. *Clin Pharmacokinet*, 2005. **44**(7):661-80.
52. Mahmood, I., C. Sahajwalla: **Clinical pharmacokinetics and pharmacodynamics of buspirone, an anxiolytic drug**. *Clin Pharmacokinet*, 1999. **36**(4):277-87.
53. Deleu, D., M.G. Northway, Y. Hanssens: **Clinical pharmacokinetic and pharmacodynamic properties of drugs used in the treatment of Parkinson's disease**. *Clin Pharmacokinet*, 2002. **41**(4):261-309.
54. Oliveira, C.H., J. Salmon, M. Sucupira, J. Ilha, G. De Nucci: **Comparative bioavailability of two cefadroxil formulations in healthy human volunteers after a single-dose administration**. *Biopharm Drug Dispos*, 2000. **21**(6):243-7.
55. Otoom, S., M. Hasan, N. Najib: **Comparative bioavailability of two cefadroxil products using serum and urine data in healthy human volunteers**. *Clin Exp Pharmacol Physiol*, 2004. **31**(7):433-7.
56. Perry, C.M., L.J. Scott: **Cefdinir: a review of its use in the management of mild-to-moderate bacterial infections**. *Drugs*, 2004. **64**(13):1433-64.
57. Klepser, M.E., M.N. Marangos, K.B. Patel, D.P. Nicolau, R. Quintiliani, C.H. Nightingale: **Clinical pharmacokinetics of newer cephalosporins**. *Clin Pharmacokinet*, 1995. **28**(5):361-84.
58. Lal, J., O.P. Asthana, S. Nityanand, R.C. Gupta: **Pharmacokinetics of centchroman in healthy female subjects after oral administration**. *Contraception*, 1995. **52**(5):297-300.
59. Veenendaal, J.R., M.D. Edstein, K.H. Rieckmann: **Pharmacokinetics of chlorproguanil in man after a single oral dose of Lapudrine**. *Chemotherapy*, 1988. **34**(4):275-83.

60. Walle, T., Y. Otake, J.A. Brubaker, U.K. Walle, P.V. Halushka: **Disposition and metabolism of the flavonoid chrysin in normal volunteers**. *Br J Clin Pharmacol,* 2001. **51**(2):143-6.
61. Hildebrand, M., T. Staks, A. Schutt, H. Matthes: **Pharmacokinetics of 3H-cicaprost in healthy volunteers**. *Prostaglandins*, 1989. **37**(2):259-73.
62. Hildebrand, M., T. Staks, B. Nieuweboer: **Pharmacokinetics and pharmacodynamics of cicaprost in healthy volunteers after oral administration of 5 to 20 micrograms**. *Eur J Clin Pharmacol,* 1990. **39**(2):149-53.
63. Gross, V., E. Treher, K. Haag, W. Neis, U. Wiegand, J. Scholmerich: **Angiotensin-converting enzyme (ACE)-inhibition in cirrhosis. Pharmacokinetics and dynamics of the ACE-inhibitor cilazapril (Ro 31-2848)**. *J Hepatol,* 1993. **17**(1):40-7.
64. Takamatsu, N., L.S. Welage, Y. Hayashi, R. Yamamoto, J.L. Barnett, V.P. Shah, L.J. Lesko, C. Ramachandran, G.L. Amidon: **Variability in cimetidine absorption and plasma double peaks following oral administration in the fasted state in humans: correlation with antral gastric motility**. *Eur J Pharm Biopharm,* 2002. **53**(1):37-47.
65. Danesi, R., M. Ducci, D. Acerbi, M. Del Tacca: **Plasma pharmacokinetics of cinmetacin following oral administration in healthy volunteers**. *Arzneimittelforschung*, 1988. **38**(1):129-31.
66. Nix, D.E., R.D. Adam, B. Auclair, T.S. Krueger, P.G. Godo, C.A. Peloquin: **Pharmacokinetics and relative bioavailability of clofazimine in relation to food, orange juice and antacid**. *Tuberculosis (Edinb),* 2004. **84**(6):365-73.
67. Venkatesan, K.: **Clinical pharmacokinetic considerations in the treatment of patients with leprosy**. *Clin Pharmacokinet,* 1989. **16**(6):365-86.
68. Mohammed, S.S., M. Ayass, P. Mehta, A. Kedar, S. Gross, H. Derendorf: **Codeine disposition in sickle cell patients compared with healthy volunteers**. *J Clin Pharmacol*, 1993. **33**(9):811-5.
69. Achtert, G., J.M. Scherrmann, M.O. Christen: **Pharmacokinetics/bioavailability of colchicine in healthy male volunteers**. *Eur J Drug Metab Pharmacokinet*, 1989. **14**(4):317-22.
70. Thomas, G., C. Girre, J.M. Scherrmann, P. Francheteau, J.L. Steimer: **Zero-order absorption and linear disposition of oral colchicine in healthy volunteers**. *Eur J Clin Pharmacol,* 1989. **37**(1):79-84.
71. Ritschel, W.A., M.E. Brady, H.S. Tan, K.A. Hoffmann, I.M. Yiu, K.W. Grummich: **Pharmacokinetics of coumarin and its 7-hydroxy-metabolites upon intravenous and peroral administration of coumarin in man**. *Eur J Clin Pharmacol,* 1977. **12**(6):457-61.
72. Zhu, M., D.E. Nix, R.D. Adam, J.M. Childs, C.A. Peloquin: **Pharmacokinetics of cycloserine under fasting conditions and with high-fat meal, orange juice, and antacids**. *Pharmacotherapy*, 2001. **21**(8):891-7.
73. Morse, G.D., M.A. Fischl, M.J. Shelton, S.R. Cox, M. Driver, M. DeRemer, W.W. Freimuth: **Single-dose pharmacokinetics of delavirdine mesylate and didanosine in patients with human immunodeficiency virus infection**. *Antimicrob Agents Chemother*, 1997. **41**(1):169-74.

74. Gupta, S., C. Banfield, M. Affrime, A. Marco, M. Cayen, J. Herron, D. Padhi: **Desloratadine demonstrates dose proportionality in healthy adults after single doses**. *Clin Pharmacokinet*, 2002. **41 Suppl 1**:1-6.
75. Pagani, I., N. Barzaghi, F. Crema, E. Perucca, D. Ego, V. Rovei: **Pharmacokinetics of dextromoramide in surgical patients**. *Fundam Clin Pharmacol*, 1989. **3**(1):27-35.
76. Zhou, X.J., L.B. Sheiner, R.T. D'Aquila, M.D. Hughes, M.S. Hirsch, M.A. Fischl, V.A. Johnson, M. Myers, J.P. Sommadossi: **Population pharmacokinetics of nevirapine, zidovudine, and didanosine in human immunodeficiency virus-infected patients. The National Institute of Allergy and Infectious Diseases AIDS Clinical Trials Group Protocol 241 Investigators**. *Antimicrob Agents Chemother*, 1999. **43**(1):121-8.
77. Bolla, S., R.R. Boinpally, S. Poondru, R. Devaraj, B.R. Jasti: **Pharmacokinetics of diethylcarbamazine after single oral dose at two different times of day in human subjects**. *J Clin Pharmacol*, 2002. **42**(3):327-31.
78. Shenoy, R.K., T.K. Suma, A. John, S.R. Arun, V. Kumaraswami, L.L. Fleckenstein, K. Na-Bangchang: **The pharmacokinetics, safety and tolerability of the co-administration of diethylcarbamazine and albendazole**. *Ann Trop Med Parasitol*, 2002. **96**(6):603-14.
79. Kirch, W., E.E. Ohnhaus, J. Pabst, L. Storstein: **Digitoxin in patients with hepatorenal insufficiency after repeated oral administration**. *Eur Heart J*, 1989. **10**(1):40-7.
80. Awni, W.M., Z. Hussein, J.H. Cavanaugh, G.R. Granneman, L.M. Dube: **Assessment of the pharmacokinetic interaction between zileuton and digoxin in humans**. *Clin Pharmacokinet*, 1995. **29 Suppl 2**:92-7.
81. Ammon, S., U. Hofmann, E.U. Griese, N. Gugeler, G. Mikus: **Pharmacokinetics of dihydrocodeine and its active metabolite after single and multiple oral dosing**. *Br J Clin Pharmacol*, 1999. **48**(3):317-22.
82. Levy, M., E. Zylber-Katz, B. Rosenkranz: **Clinical pharmacokinetics of dipyrone and its metabolites**. *Clin Pharmacokinet*, 1995. **28**(3):216-34.
83. Krall, W.J., J.J. Sramek, N.R. Cutler: **Cholinesterase inhibitors: a therapeutic strategy for Alzheimer disease**. *Ann Pharmacother*, 1999. **33**(4):441-50.
84. Lu, Y., H. Wen, W. Li, Y. Chi, Z. Zhang: **Determination of donepezil hydrochloride (E2020) in plasma by liquid chromatography-mass spectrometry and its application to pharmacokinetic studies in healthy, young, Chinese subjects**. *J Chromatogr Sci*, 2004. **42**(5):234-7.
85. Maguire, K.P., G.D. Burrows, T.R. Norman, B.A. Scoggins: **Metabolism and pharmacokinetics of dothiepin**. *Br J Clin Pharmacol*, 1981. **12**(3):405-9.
86. Meyer-Barner, M., I. Meineke, K.H. Schreeb, C.H. Gleiter: **Pharmacokinetics of doxepin and desmethyldoxepin: an evaluation with the population approach**. *Eur J Clin Pharmacol*, 2002. **58**(4):253-7.
87. Friedman, H., D.J. Greenblatt, J.M. Scavone, E.S. Burstein, H.R. Ochs, J.S. Harmatz, R.I. Shader: **Clearance of the antihistamine doxylamine. Reduced in elderly men but not in elderly women**. *Clin Pharmacokinet*, 1989. **16**(5):312-6.

88. John, B.A., R.R. Brodie, G.A. Baldock, A. McBurney, L.F. Chasseaud, P. Jank, A. Von Nieciecki: **Pharmacokinetics and metabolism of the anti-oestrogen droloxifene in female human subjects**. *Xenobiotica*, 2002. **32**(8):699-713.
89. Skinner, M.H., H.Y. Kuan, A. Skerjanec, M.E. Seger, M. Heathman, L. O'Brien, S. Reddy, M.P. Knadler: **Effect of age on the pharmacokinetics of duloxetine in women**. *Br J Clin Pharmacol*, 2004. **57**(1):54-61.
90. Stablein, J.J., S.S. Samaan, S.C. Bukantz, R.F. Lockey: **Pharmacokinetics and bioavailability of three dyphylline preparations**. *Eur J Clin Pharmacol,* 1983. **25**(2):281-3.
91. Kappelhoff, B.S., A.D. Huitema, Z. Yalvac, J.M. Prins, J.W. Mulder, P.L. Meenhorst, J.H. Beijnen: **Population pharmacokinetics of efavirenz in an unselected cohort of HIV-1-infected individuals**. *Clin Pharmacokinet,* 2005. **44**(8):849-61.
92. Lee, M.J., P. Maliakal, L. Chen, X. Meng, F.Y. Bondoc, S. Prabhu, G. Lambert, S. Mohr, C.S. Yang: **Pharmacokinetics of tea catechins after ingestion of green tea and (-)-epigallocatechin-3-gallate by humans: formation of different metabolites and individual variability**. *Cancer Epidemiol Biomarkers Prev,* 2002. **11**(10 Pt 1):1025-32.
93. Frampton, J.E., C.M. Perry: **Emtricitabine: a review of its use in the management of HIV infection**. *Drugs*, 2005. **65**(10):1427-48.
94. Lima, J.J., C.V. Leier, L. Holtz, J. Sterechele, B.J. Shields, J.J. MacKichan: **Oral enoximone pharmacokinetics in patients with congestive heart failure**. *J Clin Pharmacol*, 1987. **27**(9):654-60.
95. Hosie, J., A.K. Scott, J.C. Petrie, I.D. Cockshott: **Pharmacokinetics of epanolol after acute and chronic oral dosing in elderly patients with stable angina pectoris**. *Br J Clin Pharmacol*, 1990. **29**(3):333-7.
96. Sarashina, A., S. Tatami, N. Yamamura, Y. Tsuda, T. Igarashi: **Population pharmacokinetics of epinastine, a histamine H1 receptor antagonist, in adults and children**. *Br J Clin Pharmacol*, 2005. **59**(1):43-53.
97. Tenero, D.M., D.E. Martin, A.K. Miller, B. Ilson, S.C. Boike, N. Zariffa, D.K. Jorkasky: **Effect of age and gender on the pharmacokinetics of eprosartan**. *Br J Clin Pharmacol*, 1998. **46**(3):267-70.
98. de Groot, A.N., T.B. Vree, Y.A. Hekster, M. van den Biggelaar-Martea, P.W. van Dongen, J. van Roosmalen: **Pharmacokinetics and bioavailability of oral ergometrine in male volunteers**. *Biopharm Drug Dispos,* 1994. **15**(1):65-73.
99. Sanders, S.W., N. Haering, H. Mosberg, H. Jaeger: **Pharmacokinetics of ergotamine in healthy volunteers following oral and rectal dosing**. *Eur J Clin Pharmacol*, 1986. **30**(3):331-4.
100. Goldzieher, J.W., S.A. Brody: **Pharmacokinetics of ethinyl estradiol and mestranol**. *Am J Obstet Gynecol,* 1990. **163**(6 Pt 2):2114-9.
101. Rodrigues, A.D., R.A. Halpin, L.A. Geer, D. Cui, E.J. Woolf, C.Z. Matthews, K.M. Gottesdiener, P.J. Larson, K.C. Lasseter, N.G. Agrawal: **Absorption, metabolism, and excretion of etoricoxib, a potent and selective cyclooxygenase-2 inhibitor, in healthy male volunteers**. *Drug Metab Dispos*, 2003. **31**(2):224-32.
102. Echizen, H., T. Ishizaki: **Clinical pharmacokinetics of famotidine**. *Clin Pharmacokinet*, 1991. **21**(3):178-94.

103. Hochhaus, G., H. Mollmann: **Pharmacokinetic/pharmacodynamic characteristics of the beta-2-agonists terbutaline, salbutamol and fenoterol**. *Int J Clin Pharmacol Ther Toxicol*, 1992. **30**(9):342-62.
104. Berry, D.J., M. Webley, R. Grahame, R. Goulding, M. Gaetani, D.V. Parke: **Pharmacokinetics of single oral doses of feprazone in patients with rheumatoid arthritis or with impaired renal clearance**. *Xenobiotica*, 1993. **23**(11):1231-40.
105. Molimard, M., B. Diquet, M.S. Benedetti: **Comparison of pharmacokinetics and metabolism of desloratadine, fexofenadine, levocetirizine and mizolastine in humans**. *Fundam Clin Pharmacol*, 2004. **18**(4):399-411.
106. Dresser, G.K., R.B. Kim, D.G. Bailey: **Effect of grapefruit juice volume on the reduction of fexofenadine bioavailability: possible role of organic anion transporting polypeptides**. *Clin Pharmacol Ther*, 2005. **77**(3):170-7.
107. Elkind, A.H., A. Wade, G. Ishkanian: **Pharmacokinetics of frovatriptan in adolescent migraineurs**. *J Clin Pharmacol*, 2004. **44**(10):1158-65.
108. Taburet, A.M., J. Guibert, M.D. Kitzis, H. Sorensen, J.F. Acar, E. Singlas: **Pharmacokinetics of sodium fusidate after single and repeated infusions and oral administration of a new formulation**. *J Antimicrob Chemother*, 1990. **25 Suppl B**:23-31.
109. Boyd, R.A., D. Turck, R.B. Abel, A.J. Sedman, H.N. Bockbrader: **Effects of age and gender on single-dose pharmacokinetics of gabapentin**. *Epilepsia*, 1999. **40**(4):474-9.
110. Elwes, R.D., C.D. Binnie: **Clinical pharmacokinetics of newer antiepileptic drugs. Lamotrigine, vigabatrin, gabapentin and oxcarbazepine**. *Clin Pharmacokinet*, 1996. **30**(6):403-15.
111. Zhao, Q., M. Brett, N. Van Osselaer, F. Huang, A. Raoult, A. Van Peer, T. Verhaeghe, R. Hust: **Galantamine pharmacokinetics, safety, and tolerability profiles are similar in healthy Caucasian and Japanese subjects**. *J Clin Pharmacol*, 2002. **42**(9):1002-10.
112. Zhao, Q., G.R. Iyer, T. Verhaeghe, L. Truyen: **Pharmacokinetics and safety of galantamine in subjects with hepatic impairment and healthy volunteers**. *J Clin Pharmacol*, 2002. **42**(4):428-36.
113. Gross, A.S., M. Eichelbaum, K. Morike, G. Mikus: **Pharmacokinetics and pharmacodynamics of R- and S-gallopamil during multiple dosing**. *Br J Clin Pharmacol*, 2000. **49**(2):132-8.
114. Gross, A.S., G. Mikus, D. Ratge, H. Wisser, M. Eichelbaum: **Pharmacokinetics and pharmacodynamics of the enantiomers of gallopamil**. *J Pharmacol Exp Ther*, 1997. **281**(3):1102-12.
115. Mouly, S., G. Aymard, J.P. Tillement, C. Caulin, J.F. Bergmann, S. Urien: **Increased oral ganciclovir bioavailability in HIV-infected patients with chronic diarrhoea and wasting syndrome--a population pharmacokinetic study**. *Br J Clin Pharmacol*, 2001. **51**(6):557-65.
116. Swaisland, H.C., R.P. Smith, A. Laight, D.J. Kerr, M. Ranson, C.H. Wilder-Smith, T. Duvauchelle: **Single-dose clinical pharmacokinetic studies of gefitinib**. *Clin Pharmacokinet*, 2005. **44**(11):1165-77.

117. Kuhnz, W.: **Pharmacokinetics of the contraceptive steroids levonorgestrel and gestodene after single and multiple oral administration to women**. *Am J Obstet Gynecol*, 1990. **163**(6 Pt 2):2120-7.
118. Tauber, U., J.W. Tack, H. Matthes: **Single dose pharmacokinetics of gestodene in women after intravenous and oral administration**. *Contraception*, 1989. **40**(4):461-79.
119. Gerrits, C.J., J.H. Schellens, G.J. Creemers, P. Wissel, A.S. Planting, J.F. Pritchard, S. DePee, M. de Boer-Dennert, M. Harteveld, J. Verweij: **The bioavailability of oral GI147211 (GG211), a new topoisomerase I inhibitor**. *Br J Cancer*, 1997. **76**(7):946-51.
120. Kradjan, W.A., K.Y. Takeuchi, K.E. Opheim, F.C. Wood, Jr.: **Pharmacokinetics and pharmacodynamics of glipizide after once-daily and divided doses**. *Pharmacotherapy*, 1995. **15**(4):465-71.
121. Ali-Melkkila, T., T. Kaila, J. Kanto: **Glycopyrrolate: pharmacokinetics and some pharmacodynamic findings**. *Acta Anaesthesiol Scand*, 1989. **33**(6):513-7.
122. Chandler, M.H., S.R. Scott, R.A. Blouin: **Age-associated stereoselective alterations in hexobarbital metabolism**. *Clin Pharmacol Ther*, 1988. **43**(4):436-41.
123. Jovanovic, D., M. Maksimovic, D. Joksovic, V. Kovacevic: **Oral forms of the oxime HI-6: a study of pharmacokinetics and tolerance after administration to healthy volunteers**. *Vet Hum Toxicol*, 1990. **32**(5):419-21.
124. Schwab, M., U. Klotz: **Pharmacokinetic considerations in the treatment of inflammatory bowel disease**. *Clin Pharmacokinet*, 2001. **40**(10):723-51.
125. Schulz, H.U., M. Schurer, D. Bassler, D. Weiser: **Investigation of the bioavailability of hypericin, pseudohypericin, hyperforin and the flavonoids quercetin and isorhamnetin following single and multiple oral dosing of a hypericum extract containing tablet**. *Arzneimittelforschung*, 2005. **55**(1):15-22.
126. Kerb, R., J. Brockmoller, B. Staffeldt, M. Ploch, I. Roots: **Single-dose and steady-state pharmacokinetics of hypericin and pseudohypericin**. *Antimicrob Agents Chemother*, 1996. **40**(9):2087-93.
127. Davies, N.M.: **Clinical pharmacokinetics of ibuprofen. The first 30 years**. *Clin Pharmacokinet*, 1998. **34**(2):101-54.
128. Camaggi, C.M., E. Strocchi, P. Carisi, A. Martoni, A. Tononi, M. Guaraldi, M. Strolin-Benedetti, C. Efthymiopoulos, F. Pannuti: **Idarubicin metabolism and pharmacokinetics after intravenous and oral administration in cancer patients: a crossover study**. *Cancer Chemother Pharmacol*, 1992. **30**(4):307-16.
129. Muir, N.C., J.G. Lloyd-Jones, J.D. Nichols, J.M. Clifford: **The pharmacokinetics after intravenous and oral administration in man of the alpha 2-adrenoreceptor antagonist idazoxan (RX781094)**. *Eur J Clin Pharmacol*, 1986. **29**(6):743-5.
130. Peng, B., C. Dutreix, G. Mehring, M.J. Hayes, M. Ben-Am, M. Seiberling, R. Pokorny, R. Capdeville, P. Lloyd: **Absolute bioavailability of imatinib (Glivec) orally versus intravenous infusion**. *J Clin Pharmacol*, 2004. **44**(2):158-62.
131. Dutreix, C., B. Peng, G. Mehring, M. Hayes, R. Capdeville, R. Pokorny, M. Seiberling: **Pharmacokinetic interaction between ketoconazole and imatinib mesylate (Glivec) in healthy subjects**. *Cancer Chemother Pharmacol*, 2004. **54**(4):290-4.

132. Peng, B., P. Lloyd, H. Schran: **Clinical pharmacokinetics of imatinib**. *Clin Pharmacokinet*, 2005. **44**(9):879-94.
133. Hrdina, P.D., V. Rovei, J.F. Henry, M.P. Hervy, R. Gomeni, F. Forette, P.L. Morselli: **Comparison of single-dose pharmacokinetics of imipramine and maprotiline in the elderly**. *Psychopharmacology (Berl)*, 1980. **70**(1):29-34.
134. Guay, D.R.: **Clinical pharmacokinetics of drugs used to treat urge incontinence**. *Clin Pharmacokinet*, 2003. **42**(14):1243-85.
135. Wilson, H., M.L. Rocci, Jr., K.T. Weber, V. Andrews, M.J. Likoff: **Pharmacokinetics and hemodynamics of amrinone in patients with chronic cardiac failure of diverse etiology**. *Res Commun Chem Pathol Pharmacol*, 1987. **56**(1):3-19.
136. Vachharajani, N.N., W.C. Shyu, R.A. Smith, D.S. Greene: **The effects of age and gender on the pharmacokinetics of irbesartan**. *Br J Clin Pharmacol*, 1998. **46**(6):611-3.
137. Abshagen, U., G. Betzien, R. Endele, B. Kaufmann, G. Neugebauer: **Pharmacokinetics and metabolism of isosorbide-dinitrate after intravenous and oral administration**. *Eur J Clin Pharmacol*, 1985. **27**(6):637-44.
138. Kolle, E.U., K.O. Vollmer: **Pharmacokinetics of isoxicam following intravenous, intramuscular, oral and rectal administration in healthy volunteers**. *Br J Clin Pharmacol*, 1986. **22 Suppl 2**:135S-141S.
139. Caille, G., P. Du Souich, L. Lariviere, M. Vezina, Y. Lacasse: **The effect of administration of phenytoin on the pharmacokinetics of isoxicam**. *Biopharm Drug Dispos*, 1987. **8**(1):57-61.
140. Clifton, G.D., R.A. Blouin, C. Dilea, H.F. Schran, A.E. Hassell, L.M. Gonasun, T.S. Foster: **The pharmacokinetics of oral isradipine in normal volunteers**. *J Clin Pharmacol*, 1988. **28**(1):36-42.
141. Dennis, M.J., P.C. French, P. Crome, M. Babiker, J. Shillingford, R. Hopkins: **Pharmacokinetic profile of controlled release ketoprofen in elderly patients**. *Br J Clin Pharmacol*, 1985. **20**(6):567-73.
142. Chen, X., D. Zhong, D. Liu, Y. Wang, Y. Han, J. Gu: **Determination of ketotifen and its conjugated metabolite in human plasma by liquid chromatography/tandem mass spectrometry: application to a pharmacokinetic study**. *Rapid Commun Mass Spectrom*, 2003. **17**(22):2459-63.
143. MacCarthy, E.P., S.S. Bloomfield: **Labetalol: a review of its pharmacology, pharmacokinetics, clinical uses and adverse effects**. *Pharmacotherapy*, 1983. **3**(4):193-219.
144. Johnson, J.A., W.S. Akers, V.L. Herring, M.S. Wolfe, J.M. Sullivan: **Gender differences in labetalol kinetics: importance of determining stereoisomer kinetics for racemic drugs**. *Pharmacotherapy*, 2000. **20**(6):622-8.
145. Lalonde, R.L., T.L. O'Rear, I.W. Wainer, K.D. Drda, V.L. Herring, M.B. Bottorff: **Labetalol pharmacokinetics and pharmacodynamics: evidence of stereoselective disposition**. *Clin Pharmacol Ther*, 1990. **48**(5):509-19.
146. Chan, V., B.G. Charles, S.E. Tett: **Population pharmacokinetics and association between A77 1726 plasma concentrations and disease activity measures following administration of leflunomide to people with rheumatoid arthritis**. *Br J Clin Pharmacol*, 2005. **60**(3):257-64.

147. Jabor, V.A., E.B. Coelho, V.L. Lanchote: **Enantioselective pharmacokinetics of lercanidipine in healthy volunteers**. *J Chromatogr B Analyt Technol Biomed Life Sci*, 2004. **813**(1-2):343-6.
148. Pfister, C.U., A. Martoni, C. Zamagni, G. Lelli, F. De Braud, C. Souppart, M. Duval, U. Hornberger: **Effect of age and single versus multiple dose pharmacokinetics of letrozole (Femara) in breast cancer patients**. *Biopharm Drug Dispos*, 2001. **22**(5):191-7.
149. Zittoun, J., A.P. Tonelli, J. Marquet, E. De Gialluly, C. Hancock, A. Yacobi, J.B. Johnson: **Pharmacokinetic comparison of leucovorin and levoleucovorin**. *Eur J Clin Pharmacol*, 1993. **44**(6):569-73.
150. Kook, K., H. Gabelnick, G. Duncan: **Pharmacokinetics of levonorgestrel 0.75 mg tablets**. *Contraception*, 2002. **66**(1):73-6.
151. Sandell, E.P., M. Hayha, S. Antila, P. Heikkinen, P. Ottoila, L.A. Lehtonen, P.J. Pentikainen: **Pharmacokinetics of levosimendan in healthy volunteers and patients with congestive heart failure**. *J Cardiovasc Pharmacol,* 1995. **26 Suppl 1**:S57-62.
152. Vigushin, D.M., G.K. Poon, A. Boddy, J. English, G.W. Halbert, C. Pagonis, M. Jarman, R.C. Coombes: **Phase I and pharmacokinetic study of D-limonene in patients with advanced cancer. Cancer Research Campaign Phase I/II Clinical Trials Committee**. *Cancer Chemother Pharmacol*, 1998. **42**(2):111-7.
153. Evans, J.L., C.J. Heymann, I.D. Goldfine, L.A. Gavin: **Pharmacokinetics, tolerability, and fructosamine-lowering effect of a novel, controlled-release formulation of alpha-lipoic acid**. *Endocr Pract*, 2002. **8**(1):29-35.
154. Teichert, J., R. Hermann, P. Ruus, R. Preiss: **Plasma kinetics, metabolism, and urinary excretion of alpha-lipoic acid following oral administration in healthy volunteers**. *J Clin Pharmacol*, 2003. **43**(11):1257-67.
155. Teichert, J., J. Kern, H.J. Tritschler, H. Ulrich, R. Preiss: **Investigations on the pharmacokinetics of alpha-lipoic acid in healthy volunteers**. *Int J Clin Pharmacol Ther*, 1998. **36**(12):625-8.
156. Oki, T., Y. Usami, M. Nakai, M. Sagisaka, H. Ito, K. Nagaoka, N. Mamiya, K. Yamanaka, M. Utsumi, T. Kaneda: **Pharmacokinetics of lopinavir after administration of Kaletra in healthy Japanese volunteers**. *Biol Pharm Bull*, 2004. **27**(2):261-5.
157. Yin, O.Q., X.J. Shi, B. Tomlinson, M.S. Chow: **Effect of cyp2d6*10 allele on the pharmacokinetics of loratadine in chinese subjects**. *Drug Metab Dispos*, 2005. **33**(9):1283-7.
158. Tanumihardjo, S.A., J. Li, M.P. Dosti: **Lutein absorption is facilitated with cosupplementation of ascorbic acid in young adults**. *J Am Diet Assoc*, 2005. **105**(1):114-8.
159. Buhrer, M., J.Y. Le Cotonnec, M. Wermeille, J. Bircher: **Treatment of liver disease with malotilate. A pharmacokinetic and pharmacodynamic phase II study in cirrhosis**. *Eur J Clin Pharmacol*, 1986. **30**(4):407-16.
160. Dawson, M., P.A. Braithwaite, M.S. Roberts, T.R. Watson: **The pharmacokinetics and bioavailability of a tracer dose of [3H]-mebendazole in man**. *Br J Clin Pharmacol*, 1985. **19**(1):79-86.

161. Boulton-Jones, J.M., C.G. Geddes, G. Heinzel, D. Turck, G. Nehmiz, P.J. Bevis: **Meloxicam pharmacokinetics in renal impairment**. *Br J Clin Pharmacol*, 1997. **43**(1):35-40.
162. Birnie, G.G., G.G. Thompson, T. Murray, G. Watkinson, M.J. Brodie: **Enhanced oral bioavailability of meptazinol in cirrhosis**. *Gut*, 1987. **28**(3):248-54.
163. Murray, G.R., O. Petitjean, R.A. Franklin, D.F. Graham, J.H. Trouvin, C. Jacquot: **The systemic availability of meptazinol in man after oral and rectal doses**. *Eur J Clin Pharmacol*, 1989. **36**(3):279-82.
164. Norbury, H.M., R.A. Franklin, D.F. Graham, B. Sinha: **Pharmacokinetics of meptazinol after single and multiple oral administration to elderly patients**. *Eur J Clin Pharmacol*, 1984. **27**(2):223-6.
165. Bostrom, B., G. Erdmann: **Cellular pharmacology of 6-mercaptopurine in acute lymphoblastic leukemia**. *Am J Pediatr Hematol Oncol*, 1993. **15**(1):80-6.
166. Taft, D.R., S. Nordt, G.R. Iyer, M.H. Schwenk: **Blood disposition and urinary excretion kinetics of methazolamide following oral administration to human subjects**. *Biopharm Drug Dispos*, 1998. **19**(6):373-80.
167. Haagsma, C.J., F.G. Russel, T.B. Vree, P.L. Van Riel, L.B. Van de Putte: **Combination of methotrexate and sulphasalazine in patients with rheumatoid arthritis: pharmacokinetic analysis and relationship to clinical response**. *Br J Clin Pharmacol*, 1996. **42**(2):195-200.
168. Sinnett, M.J., G.D. Groff, D.A. Raddatz, W.A. Franck, J.S. Bertino, Jr.: **Methotrexate pharmacokinetics in patients with rheumatoid arthritis**. *J Rheumatol*, 1989. **16**(6):745-8.
169. Dahl, S.G., R.E. Strandjord, S. Sigfusson: **Pharmacokinetics and relative bioavailability of levomepromazine after repeated administration of tablets and syrup**. *Eur J Clin Pharmacol*, 1977. **11**(4):305-10.
170. Campbell, N.R., A. Skerjanec, Y. Tam, S. Robertson, E. Burgess: **Methyldopa kinetics before and after ingestion of methyldopa for eight weeks**. *Eur J Clin Pharmacol*, 1995. **48**(5):397-400.
171. Edsbacker, S., T. Andersson: **Pharmacokinetics of budesonide (Entocort EC) capsules for Crohn's disease**. *Clin Pharmacokinet*, 2004. **43**(12):803-21.
172. Cerqueira, P.M., E.J. Cesarino, F.H. Mateus, Y. Mere, Jr., S.R. Santos, V.L. Lanchote: **Enantioselectivity in the steady-state pharmacokinetics of metoprolol in hypertensive patients**. *Chirality*, 1999. **11**(7):591-7.
173. Grech-Belanger, O., G. Barbeau, P. Kishka, C. Fiset, E. LeBoeuf, M. Blouin: **Pharmacokinetics of mexiletine in the elderly**. *J Clin Pharmacol*, 1989. **29**(4):311-5.
174. Mehvar, R., D.R. Brocks, M. Vakily: **Impact of stereoselectivity on the pharmacokinetics and pharmacodynamics of antiarrhythmic drugs**. *Clin Pharmacokinet*, 2002. **41**(8):533-58.
175. Uenaka, K., T. Koue, T. Iwai, M. Shibakawa, K. Ueno: **Population pharmacokinetic analysis of mexiletine in adult arrhythmic patients in Japanese population**. *Biol Pharm Bull*, 1998. **21**(8):844-6.
176. Jabor, V.A., E.B. Coelho, N.A. Dos Santos, P.S. Bonato, V.L. Lanchote: **A highly sensitive LC-MS-MS assay for analysis of midazolam and its major metabolite in**

human plasma: applications to drug metabolism. *J Chromatogr B Analyt Technol Biomed Life Sci*, 2005. **822**(1-2):27-32.
177. Imbimbo, B.P., M. Seiberling, U. Peuckert, G. Hoexter, H. Maier-Lenz, A. Vidi, S. Daniotti: **Safety and pharmacokinetics of mifentidine after increasing oral doses in healthy subjects**. *Eur J Clin Pharmacol*, 1988. **35**(6):673-6.
178. Liu, J.H., V.G. Garzo, S.S. Yen: **Pharmacodynamics of the antiprogesterone RU486 in women after oral administration**. *Fertil Steril*, 1988. **50**(2):245-9.
179. Saivin, S., G. Houin: **Clinical pharmacokinetics of doxycycline and minocycline**. *Clin Pharmacokinet*, 1988. **15**(6):355-66.
180. Fleishaker, J.C., N.A. Andreadis, I.R. Welshman, C.E. Wright, 3rd: **The pharmacokinetics of 2.5- to 10-mg oral doses of minoxidil in healthy volunteers**. *J Clin Pharmacol*, 1989. **29**(2):162-7.
181. Kirchheiner, J., H.B. Henckel, I. Meineke, I. Roots, J. Brockmoller: **Impact of the CYP2D6 ultrarapid metabolizer genotype on mirtazapine pharmacokinetics and adverse events in healthy volunteers**. *J Clin Psychopharmacol*, 2004. **24**(6):647-52.
182. Wong, Y.N., S.P. King, W.B. Laughton, G.C. McCormick, P.E. Grebow: **Single-dose pharmacokinetics of modafinil and methylphenidate given alone or in combination in healthy male volunteers**. *J Clin Pharmacol*, 1998. **38**(3):276-82.
183. Rosenkranz, B., B.R. Winkelmann, M.J. Parnham: **Clinical pharmacokinetics of molsidomine**. *Clin Pharmacokinet*, 1996. **30**(5):372-84.
184. Graff, G.R., A. Weber, D. Wessler-Starman, A.L. Smith: **Montelukast pharmacokinetics in cystic fibrosis**. *J Pediatr*, 2003. **142**(1):53-6.
185. Penson, R.T., S.P. Joel, M. Roberts, A. Gloyne, S. Beckwith, M.L. Slevin: **The bioavailability and pharmacokinetics of subcutaneous, nebulized and oral morphine-6-glucuronide**. *Br J Clin Pharmacol*, 2002. **53**(4):347-54.
186. Bullingham, R.E., A.J. Nicholls, B.R. Kamm: **Clinical pharmacokinetics of mycophenolate mofetil**. *Clin Pharmacokinet*, 1998. **34**(6):429-55.
187. Le Guellec, C., H. Bourgoin, M. Buchler, Y. Le Meur, Y. Lebranchu, P. Marquet, G. Paintaud: **Population pharmacokinetics and Bayesian estimation of mycophenolic acid concentrations in stable renal transplant patients**. *Clin Pharmacokinet*, 2004. **43**(4):253-66.
188. Aitkenhead, A.R., E.S. Lin, K.J. Achola: **The pharmacokinetics of oral and intravenous nalbuphine in healthy volunteers**. *Br J Clin Pharmacol*, 1988. **25**(2):264-8.
189. Davies, N.M., K.E. Anderson: **Clinical pharmacokinetics of naproxen**. *Clin Pharmacokinet*, 1997. **32**(4):268-93.
190. Christensen, M.L., S.K. Eades, E. Fuseau, R.D. Kempsford, S.J. Phelps, L.J. Hak: **Pharmacokinetics of naratriptan in adolescent subjects with a history of migraine**. *J Clin Pharmacol*, 2001. **41**(2):170-5.
191. Aymard, G., D. Warot, P. Demolis, J.F. Giudicelli, P. Lechat, M.E. Le Guern, C. Alquier, B. Diquet: **Comparative pharmacokinetics and pharmacodynamics of intravenous and oral nefopam in healthy volunteers**. *Pharmacol Toxicol*, 2003. **92**(6):279-86.
192. Peck, R.W., B.C. Weatherley, R. Wootton, P. Crome, T.A. Holdich, J. Posner: **Pharmacokinetics and tolerability of single oral doses of 882C87, a potent, new**

anti-varicella-zoster virus agent, in healthy volunteers. *Antimicrob Agents Chemother*, 1995. **39**(1):20-7.
193. Sabo, J.P., M.J. Lamson, G. Leitz, C.L. Yong, T.R. MacGregor: **Pharmacokinetics of nevirapine and lamivudine in patients with HIV-1 infection**. *AAPS PharmSci*, 2000. **2**(1):E1.
194. Svensson, C.K.: **Clinical pharmacokinetics of nicotine**. *Clin Pharmacokinet*, 1987. **12**(1):30-40.
195. Grundy, J.S., R.T. Foster: **The nifedipine gastrointestinal therapeutic system (GITS). Evaluation of pharmaceutical, pharmacokinetic and pharmacological properties**. *Clin Pharmacokinet*, 1996. **30**(1):28-51.
196. Renwick, A.G., D.R. Robertson, B. Macklin, V. Challenor, D.G. Waller, C.F. George: **The pharmacokinetics of oral nifedipine--a population study**. *Br J Clin Pharmacol*, 1988. **25**(6):701-8.
197. Bernareggi, A.: **Clinical pharmacokinetics of nimesulide**. *Clin Pharmacokinet*, 1998. **35**(4):247-74.
198. Muck, W., H.P. Breuel, J. Kuhlmann: **The influence of age on the pharmacokinetics of nimodipine**. *Int J Clin Pharmacol Ther*, 1996. **34**(7):293-8.
199. Chandler, M.H., G.D. Clifton, J.T. Lettieri, A.L. Mazzu, D.R. Allington, A.C. Thieneman, T.S. Foster, M.R. Harrison: **Multiple dose pharmacokinetics of four different doses of nisoldipine in hypertensive patients**. *J Clin Pharmacol*, 1992. **32**(6):571-5.
200. Abernethy, D.R., D.J. Greenblatt, A. Locniskar, H.R. Ochs, J.S. Harmatz, R.I. Shader: **Obesity effects on nitrazepam disposition**. *Br J Clin Pharmacol*, 1986. **22**(5):551-7.
201. Soons, P.A., D.D. Breimer: **Stereoselective pharmacokinetics of oral and intravenous nitrendipine in healthy male subjects**. *Br J Clin Pharmacol*, 1991. **32**(1):11-6.
202. Callaghan, J.T., R.F. Bergstrom, A. Rubin, S. Chernish, R. Crabtree, M.P. Knadler, B. Obermeyer, W.W. Offen, D.W. Schneck, G. Aronoff, et al.: **A pharmacokinetic profile of nizatidine in man**. *Scand J Gastroenterol Suppl*, 1987. **136**:9-17.
203. Aronoff, G.R., R.F. Bergstrom, R.J. Bopp, R.S. Sloan, J.T. Callaghan: **Nizatidine disposition in subjects with normal and impaired renal function**. *Clin Pharmacol Ther*, 1988. **43**(6):688-95.
204. Tserng, K.Y., S.T. Ingalls, E.M. Boczko, T.P. Spiro, X. Li, S. Majka, S.L. Gerson, J.K. Willson, C.L. Hoppel: **Pharmacokinetics of O6-benzylguanine (NSC637037) and its metabolite, 8-oxo-O6-benzylguanine**. *J Clin Pharmacol*, 2003. **43**(8):881-93.
205. Hagg, S., O. Spigset, H.A. Lakso, R. Dahlqvist: **Olanzapine disposition in humans is unrelated to CYP1A2 and CYP2D6 phenotypes**. *Eur J Clin Pharmacol*, 2001. **57**(6-7):493-7.
206. Penzak, S.R., Y.Y. Hon, W.D. Lawhorn, K.L. Shirley, V. Spratlin, M.W. Jann: **Influence of ritonavir on olanzapine pharmacokinetics in healthy volunteers**. *J Clin Psychopharmacol*, 2002. **22**(4):366-70.

207. Marier, J.F., M.C. Dubuc, E. Drouin, F. Alvarez, M.P. Ducharme, J.L. Brazier: **Pharmacokinetics of omeprazole in healthy adults and in children with gastroesophageal reflux disease**. *Ther Drug Monit*, 2004. **26**(1):3-8.
208. Roila, F., A. Del Favero: **Ondansetron clinical pharmacokinetics**. *Clin Pharmacokinet*, 1995. **29**(2):95-109.
209. Davies, N.M.: **Clinical pharmacokinetics of oxaprozin**. *Clin Pharmacokinet*, 1998. **35**(6):425-36.
210. Leucuta, S.E., M. Follidis, R. Capalneanu, A. Mocan: **Relative bioavailability of different oral sustained release oxprenolol tablets**. *Eur J Drug Metab Pharmacokinet*, 1998. **23**(2):178-84.
211. Leow, K.P., M.T. Smith, J.A. Watt, B.E. Williams, T. Cramond: **Comparative oxycodone pharmacokinetics in humans after intravenous, oral, and rectal administration**. *Ther Drug Monit*, 1992. **14**(6):479-84.
212. Poyhia, R., A. Vainio, E. Kalso: **A review of oxycodone's clinical pharmacokinetics and pharmacodynamics**. *J Pain Symptom Manage*, 1993. **8**(2):63-7.
213. Ehrnebo, M., L.O. Boreus, U. Lonroth: **Bioavailability and first-pass metabolism of oral pentazocine in man**. *Clin Pharmacol Ther*, 1977. **22**(6):888-92.
214. Ehrnebo, M.: **Pharmacokinetics and distribution properties of pentobarbital in humans following oral and intravenous administration**. *J Pharm Sci*, 1974. **63**(7):1114-8.
215. Rakhit, A., M.E. Hurley, V. Tipnis, J. Coleman, A. Rommel, H.R. Brunner: **Pharmacokinetics and pharmacodynamics of pentopril, a new angiotensin-converting-enzyme inhibitor in humans**. *J Clin Pharmacol*, 1986. **26**(3):156-64.
216. Mauro, V.F., L.S. Mauro, J.H. Hageman: **Comparison of pentoxifylline pharmacokinetics between smokers and nonsmokers**. *J Clin Pharmacol*, 1992. **32**(11):1054-8.
217. Dong, S.X., Z.Z. Ping, W.Z. Xiao, C.C. Shu, A. Bartoli, G. Gatti, S. D'Urso, E. Perucca: **Effect of active and passive cigarette smoking on CYP1A2-mediated phenacetin disposition in Chinese subjects**. *Ther Drug Monit*, 1998. **20**(4):371-5.
218. Kirchheiner, J., M. Ufer, E.C. Walter, B. Kammerer, R. Kahlich, C. Meisel, M. Schwab, C.H. Gleiter, A. Rane, I. Roots, J. Brockmoller: **Effects of CYP2C9 polymorphisms on the pharmacokinetics of R- and S-phenprocoumon in healthy volunteers**. *Pharmacogenetics*, 2004. **14**(1):19-26.
219. Sioufi, A., D. Colussi, F. Caudal, J.P. Schoeller, P. Massias: **Pharmacokinetics of phenylbutazone in healthy subjects after oral administration of single and multiple doses**. *J Pharm Sci*, 1980. **69**(12):1413-6.
220. Walter, K., M. Muller, M.F. Barkworth, A.V. Nieciecki, F. Stanislaus: **Pharmacokinetics of physostigmine in man following a single application of a transdermal system**. *Br J Clin Pharmacol*, 1995. **39**(1):59-63.
221. Chau, N.P., Y.A. Weiss, M.E. Safar, D.E. Lavene, D.R. Georges, P.L. Milliez: **Pindolol availability in hypertensive patients with normal and impaired renal function**. *Clin Pharmacol Ther*, 1977. **22**(5 Pt 1):505-10.
222. Weiss, G.R., G.A. Sarosy, T.D. Shenkenberg, T. Williams, N.J. Clendeninn, D.D. Von Hoff, J.L. Woolley, S.H. Liao, M.R. Blum: **A phase I clinical and**

pharmacological study of weekly intravenous infusions of piritrexim (BW301U). *Eur J Cancer Clin Oncol*, 1989. **25**(12):1867-73.

223. Rodriguez, C.A., N.E. Azie, G. Adams, K. Donaldson, S.F. Francom, B.A. Staton, P.A. Bombardt: **Single oral dose safety, tolerability, and pharmacokinetics of PNU-96391 in healthy volunteers**. *J Clin Pharmacol*, 2004. **44**(3):276-83.
224. Krieter, P., B. Flannery, T. Musick, M. Gohdes, M. Martinho, R. Courtney: **Disposition of posaconazole following single-dose oral administration in healthy subjects**. *Antimicrob Agents Chemother*, 2004. **48**(9):3543-51.
225. Wright, C.E., T.L. Sisson, A.K. Ichhpurani, G.R. Peters: **Steady-state pharmacokinetic properties of pramipexole in healthy volunteers**. *J Clin Pharmacol*, 1997. **37**(6):520-5.
226. Brocks, D.R., J. Upward, M. Davy, K. Howland, C. Compton, C. McHugh, M.J. Dennis: **Evening dosing is associated with higher plasma concentrations of pranlukast, a leukotriene receptor antagonist, in healthy male volunteers**. *Br J Clin Pharmacol*, 1997. **44**(3):289-91.
227. Twomey, T.M., D.C. Hobbs: **Analysis of prazosin in plasma by a sensitive high-performance liquid chromatographic-fluorescence method**. *J Pharm Sci*, 1978. **67**(10):1468-9.
228. Vincent, J., P.A. Meredith, J.L. Reid, H.L. Elliott, P.C. Rubin: **Clinical pharmacokinetics of prazosin--1985**. *Clin Pharmacokinet*, 1985. **10**(2):144-54.
229. Luippold, G., S. Schneider, M. Marto, P. Benohr, B. Muhlbauer: **Pharmacokinetics of two oral prednisolone tablet formulations in healthy volunteers**. *Arzneimittelforschung*, 2001. **51**(11):911-5.
230. Ronn, O.: **Pharmacokinetics of prenalterol in healthy subjects and patients with congestive heart failure**. *Acta Med Scand Suppl*, 1982. **659**:89-98.
231. Martines, C., G. Gatti, E. Sasso, S. Calzetti, E. Perucca: **The disposition of primidone in elderly patients**. *Br J Clin Pharmacol*, 1990. **30**(4):607-11.
232. Sato, J., Y. Sekizawa, A. Yoshida, E. Owada, N. Sakuta, M. Yoshihara, T. Goto, Y. Kobayashi, K. Ito: **Single-dose kinetics of primidone in human subjects: effect of phenytoin on formation and elimination of active metabolites of primidone, phenobarbital and phenylethylmalonamide**. *J Pharmacobiodyn*, 1992. **15**(9):467-72.
233. Hu, O.Y., H.S. Tang, T.Y. Sheeng, S.C. Chen, S.K. Lee, P.H. Chung: **Pharmacokinetics of promazine: I. Disposition in patients with acute viral hepatitis B**. *Biopharm Drug Dispos*, 1990. **11**(7):557-68.
234. Chen, X., D. Zhong, H. Blume: **Stereoselective pharmacokinetics of propafenone and its major metabolites in healthy Chinese volunteers**. *Eur J Pharm Sci*, 2000. **10**(1):11-6.
235. Korduba, C.A., J. Veals, E. Radwanski, S. Symchowicz, M. Chung: **Bioavailability of orally administered propiram fumarate in humans**. *J Pharm Sci*, 1981. **70**(5):521-3.
236. Cheymol, G., J.M. Poirier, P.A. Carrupt, B. Testa, J. Weissenburger, J.C. Levron, E. Snoeck: **Pharmacokinetics of beta-adrenoceptor blockers in obese and normal volunteers**. *Br J Clin Pharmacol*, 1997. **43**(6):563-70.

237. Ringhand, H.P., W.A. Ritschel, M.C. Meyer, A.B. Straughn, T. Hardt: **Pharmacokinetics of propylthiouracil upon p.o. administration in man**. *Int J Clin Pharmacol Ther Toxicol*, 1980. **18**(11):488-93.
238. DeVane, C.L., C.B. Nemeroff: **Clinical pharmacokinetics of quetiapine: an atypical antipsychotic**. *Clin Pharmacokinet*, 2001. **40**(7):509-22.
239. Li, K.Y., X. Li, Z.N. Cheng, W.X. Peng, B.K. Zhang, H.D. Li: **Multiple dose pharmacokinetics of quetiapine and some of its metabolites in Chinese suffering from schizophrenia**. *Acta Pharmacol Sin*, 2004. **25**(3):390-4.
240. Krishna, S., N.J. White: **Pharmacokinetics of quinine, chloroquine and amodiaquine. Clinical implications**. *Clin Pharmacokinet*, 1996. **30**(4):263-99.
241. Czock, D., F. Keller, M. Heringa, F.M. Rasche: **Raloxifene pharmacokinetics in males with normal and impaired renal function**. *Br J Clin Pharmacol*, 2005. **59**(4):479-82.
242. Jerling, M., B.L. Huan, K. Leung, N. Chu, H. Abdallah, Z. Hussein: **Studies to investigate the pharmacokinetic interactions between ranolazine and ketoconazole, diltiazem, or simvastatin during combined administration in healthy subjects**. *J Clin Pharmacol*, 2005. **45**(4):422-33.
243. Edwards, D.M., C. Pellizzoni, H.P. Breuel, A. Berardi, M.G. Castelli, E. Frigerio, I. Poggesi, M. Rocchetti, A. Dubini, M. Strolin Benedetti: **Pharmacokinetics of reboxetine in healthy volunteers. Single oral doses, linearity and plasma protein binding**. *Biopharm Drug Dispos*, 1995. **16**(6):443-60.
244. Kajosaari, L.I., M. Niemi, M. Neuvonen, J. Laitila, P.J. Neuvonen, J.T. Backman: **Cyclosporine markedly raises the plasma concentrations of repaglinide**. *Clin Pharmacol Ther*, 2005. **78**(4):388-99.
245. Laskin, O.L., J.A. Longstreth, C.C. Hart, D. Scavuzzo, C.M. Kalman, J.D. Connor, R.B. Roberts: **Ribavirin disposition in high-risk patients for acquired immunodeficiency syndrome**. *Clin Pharmacol Ther*, 1987. **41**(5):546-55.
246. Morse, G.D., M.J. Shelton, A.M. O'Donnell: **Comparative pharmacokinetics of antiviral nucleoside analogues**. *Clin Pharmacokinet*, 1993. **24**(2):101-23.
247. Musson, D.G., K.L. Birk, D.L. Panebianco, K.D. Gagliano, J.D. Rogers, M.R. Goldberg: **Pharmacokinetics of rizatriptan in healthy elderly subjects**. *Int J Clin Pharmacol Ther*, 2001. **39**(10):447-52.
248. Vyas, K.P., R.A. Halpin, L.A. Geer, J.D. Ellis, L. Liu, H. Cheng, C. Chavez-Eng, B.K. Matuszewski, S.L. Varga, A.R. Guiblin, J.D. Rogers: **Disposition and pharmacokinetics of the antimigraine drug, rizatriptan, in humans**. *Drug Metab Dispos*, 2000. **28**(1):89-95.
249. Krause, W., G. Kuhne, H. Matthes: **Pharmacokinetics of the antidepressant rolipram in healthy volunteers**. *Xenobiotica*, 1989. **19**(6):683-92.
250. Sweatman, T.W., A.G. Renwick, C.D. Burgess: **The pharmacokinetics of saccharin in man**. *Xenobiotica*, 1981. **11**(8):531-40.
251. Putcha, L., N.M. Cintron, J. Tsui, J.M. Vanderploeg, W.G. Kramer: **Pharmacokinetics and oral bioavailability of scopolamine in normal subjects**. *Pharm Res*, 1989. **6**(6):481-5.
252. Renner, U.D., R. Oertel, W. Kirch: **Pharmacokinetics and pharmacodynamics in clinical use of scopolamine**. *Ther Drug Monit*, 2005. **27**(5):655-65.

253. Anttila, M., E.A. Sotaniemi, O. Pelkonen, A. Rautio: **Marked effect of liver and kidney function on the pharmacokinetics of selegiline**. *Clin Pharmacol Ther*, 2005. **77**(1):54-62.
254. Mahmood, I.: **Clinical pharmacokinetics and pharmacodynamics of selegiline. An update**. *Clin Pharmacokinet*, 1997. **33**(2):91-102.
255. Kahela, P., M. Anttila, R. Tikkanen, H. Sundquist: **Effect of food, food constituents and fluid volume on the bioavailability of sotalol**. *Acta Pharmacol Toxicol (Copenh)*, 1979. **44**(1):7-12.
256. Dorr, M.B., R.D. Johnson, B. Jensen, D. Magner, T. Marbury, G.H. Talbot: **Pharmacokinetics of sparfloxacin in patients with renal impairment**. *Clin Ther*, 1999. **21**(7):1202-15.
257. Rana, K.Z., M.N. Dudley: **Clinical pharmacokinetics of stavudine**. *Clin Pharmacokinet*, 1997. **33**(4):276-84.
258. Oie, S., J.G. Gambertoglio, L. Fleckenstein: **Comparison of the disposition of total and unbound sulfisoxazole after single and multiple dosing**. *J Pharmacokinet Biopharm*, 1982. **10**(2):157-72.
259. Boisvert, A., G. Barbeau, P.M. Belanger: **Pharmacokinetics of sulfisoxazole in young and elderly subjects**. *Gerontology*, 1984. **30**(2):125-31.
260. Forgue, S.T., P.A. Reece, A.J. Sedman, T.M. deVries: **Inhibition of tacrine oral clearance by cimetidine**. *Clin Pharmacol Ther*, 1996. **59**(4):444-9.
261. Curran, M., G. Keating: **Tadalafil**. *Drugs*, 2003. **63**(20):2203-12; discussion 2213-4.
262. Perret, C., B. Lenfant, E. Weinling, D.H. Wessels, H.E. Scholtz, G. Montay, E. Sultan: **Pharmacokinetics and absolute oral bioavailability of an 800-mg oral dose of telithromycin in healthy young and elderly volunteers**. *Chemotherapy*, 2002. **48**(5):217-23.
263. Britten, C.D., E.K. Rowinsky, S.D. Baker, S.S. Agarwala, J.R. Eckardt, R. Barrington, S.G. Diab, L.A. Hammond, T. Johnson, M. Villalona-Calero, U. Fraass, P. Statkevich, D.D. Von Hoff, S.G. Eckhardt: **A Phase I and pharmacokinetic study of temozolomide and cisplatin in patients with advanced solid malignancies**. *Clin Cancer Res*, 1999. **5**(7):1629-37.
264. Dhodapkar, M., J. Rubin, J.M. Reid, P.A. Burch, H.C. Pitot, J.C. Buckner, M.M. Ames, V.J. Suman: **Phase I trial of temozolomide (NSC 362856) in patients with advanced cancer**. *Clin Cancer Res*, 1997. **3**(7):1093-100.
265. Marzolini, C., L.A. Decosterd, F. Shen, M. Gander, S. Leyvraz, J. Bauer, T. Buclin, J. Biollaz, F. Lejeune: **Pharmacokinetics of temozolomide in association with fotemustine in malignant melanoma and malignant glioma patients: comparison of oral, intravenous, and hepatic intra-arterial administration**. *Cancer Chemother Pharmacol*, 1998. **42**(6):433-40.
266. Day, R.O., G. Geisslinger, P. Paull, K.M. Williams: **Neither cimetidine nor probenecid affect the pharmacokinetics of tenoxicam in normal volunteers**. *Br J Clin Pharmacol*, 1994. **37**(1):79-81.
267. Nilsen, O.G.: **Clinical pharmacokinetics of tenoxicam**. *Clin Pharmacokinet*, 1994. **26**(1):16-43.

268. Kang, B.C., C.Q. Yang, J.E. Rhee, O.K. Suh, W.G. Shin: **High-performance liquid chromatographic analysis and pharmacokinetics of terazosin in healthy volunteers**. *Res Commun Mol Pathol Pharmacol*, 2001. **110**(5-6):371-7.
269. Borgstrom, L., L. Nyberg, S. Jonsson, C. Lindberg, J. Paulson: **Pharmacokinetic evaluation in man of terbutaline given as separate enantiomers and as the racemate**. *Br J Clin Pharmacol*, 1989. **27**(1):49-56.
270. Lalonde, R.L., D. Lessard, J. Gaudreault: **Population pharmacokinetics of terfenadine**. *Pharm Res*, 1996. **13**(6):832-8.
271. Hallen, B., M.O. Karlsson, S. Stromberg, B. Noren: **Bioavailability and disposition of terodiline in man**. *J Pharm Sci*, 1994. **83**(9):1241-6.
272. Tauber, U., K. Schroder, B. Dusterberg, H. Matthes: **Absolute bioavailability of testosterone after oral administration of testosterone-undecanoate and testosterone**. *Eur J Drug Metab Pharmacokinet*, 1986. **11**(2):145-9.
273. Noormohamed, F.H., M.S. Youle, C.J. Higgs, K.A. Kook, D.A. Hawkins, A.F. Lant, S.D. Thomas: **Pharmacokinetics and hemodynamic effects of single oral doses of thalidomide in asymptomatic human immunodeficiency virus-infected subjects**. *AIDS Res Hum Retroviruses*, 1999. **15**(12):1047-52.
274. Snel, S., J.A. Jansen, H.B. Mengel, A. Richens, S. Larsen: **The pharmacokinetics of tiagabine in healthy elderly volunteers and elderly patients with epilepsy**. *J Clin Pharmacol*, 1997. **37**(11):1015-20.
275. Davies, N.M.: **Clinical pharmacokinetics of tiaprofenic acid and its enantiomers**. *Clin Pharmacokinet*, 1996. **31**(5):331-47.
276. Gross, A.S., S. Bridge, G.M. Shenfield: **Pharmacokinetics of tolbutamide in ethnic Chinese**. *Br J Clin Pharmacol*, 1999. **47**(2):151-6.
277. Lloyd-Jones, J.G., R. Henson, J.D. Nichols, D. Greenslade, J.M. Clifford: **Pharmacokinetics of intravenous and oral tolmesoxide**. *Eur J Clin Pharmacol*, 1981. **19**(2):119-25.
278. Benedetti, M.S., V. Rovei, S.J. Dencker, A. Nagy, R. Johansson: **Pharmacokinetics of toloxatone in man following intravenous and oral administrations**. *Arzneimittelforschung*, 1982. **32**(3):276-80.
279. Olsson, B., J. Szamosi: **Multiple dose pharmacokinetics of a new once daily extended release tolterodine formulation versus immediate release tolterodine**. *Clin Pharmacokinet*, 2001. **40**(3):227-35.
280. Britzi, M., E. Perucca, S. Soback, R.H. Levy, C. Fattore, F. Crema, G. Gatti, D.R. Doose, B.E. Maryanoff, M. Bialer: **Pharmacokinetic and metabolic investigation of topiramate disposition in healthy subjects in the absence and in the presence of enzyme induction by carbamazepine**. *Epilepsia*, 2005. **46**(3):378-84.
281. Doose, D.R., S.A. Walker, L.G. Gisclon, R.K. Nayak: **Single-dose pharmacokinetics and effect of food on the bioavailability of topiramate, a novel antiepileptic drug**. *J Clin Pharmacol*, 1996. **36**(10):884-91.
282. Herben, V.M., W.W. ten Bokkel Huinink, J.H. Beijnen: **Clinical pharmacokinetics of topotecan**. *Clin Pharmacokinet*, 1996. **31**(2):85-102.
283. Sauer, R., A. Heuser: **[Topoisomerase I inhibitor with potential radiosensitizing effect]**. *Strahlenther Onkol*, 1997. **173**(3):125-30.
284. Knauf, H., E. Mutschler: **Clinical pharmacokinetics and pharmacodynamics of torasemide**. *Clin Pharmacokinet*, 1998. **34**(1):1-24.

285. Vormfelde, S.V., S. Engelhardt, A. Zirk, I. Meineke, F. Tuchen, J. Kirchheiner, J. Brockmoller: **CYP2C9 polymorphisms and the interindividual variability in pharmacokinetics and pharmacodynamics of the loop diuretic drug torsemide**. *Clin Pharmacol Ther*, 2004. **76**(6):557-66.
286. Pilbrant, A., M. Schannong, J. Vessman: **Pharmacokinetics and bioavailability of tranexamic acid**. *Eur J Clin Pharmacol*, 1981. **20**(1):65-72.
287. Weiss, M., W. Sziegoleit, K. Ponicke, M. Schobess, A. Fahr, H.J. Mest: **Bioavailability of trapidil tablets**. *Arzneimittelforschung*, 1989. **39**(9):1137-8.
288. Harder, S., P.A. Thurmann, A. Hellstern, A. Benjaminov: **Pharmacokinetics of trapidil, an antagonist of platelet derived growth factor, in healthy subjects and in patients with liver cirrhosis**. *Br J Clin Pharmacol*, 1996. **42**(4):443-9.
289. Regazzi, M.B., I. Iacona, C. Gervasutti, M. Lazzarino, S. Toma: **Clinical pharmacokinetics of tretinoin**. *Clin Pharmacokinet*, 1997. **32**(5):382-402.
290. Loi, C.M., E.J. Randinitis, A.B. Vassos, D.J. Kazierad, J.R. Koup, A.J. Sedman: **Lack of effect of type II diabetes on the pharmacokinetics of troglitazone in a multiple-dose study**. *J Clin Pharmacol*, 1997. **37**(12):1114-20.
291. Kees, F., L. Farber, M. Bucher, G. Mair, K. Morike, H. Grobecker: **Pharmacokinetics of therapeutic doses of tropisetron in healthy volunteers**. *Br J Clin Pharmacol*, 2001. **52**(6):705-7.
292. Doroshyenko, O., A. Jetter, K.P. Odenthal, U. Fuhr: **Clinical pharmacokinetics of trospium chloride**. *Clin Pharmacokinet*, 2005. **44**(7):701-20.
293. Teng, R., L.C. Dogolo, S.A. Willavize, H.L. Friedman, J. Vincent: **Oral bioavailability of trovafloxacin with and without food in healthy volunteers**. *J Antimicrob Chemother*, 1997. **39 Suppl B**:87-92.
294. Shepherd, A.M.: **Human pharmacology of urapidil**. *Drugs*, 1988. **35 Suppl 6**:34-9.
295. Scalia, S., R. Scagliarini, P. Pazzi: **Evaluation of ursodeoxycholic acid bioavailability from immediate- and sustained-release preparations using gas chromatography-mass spectrometry and high-performance liquid chromatography**. *Arzneimittelforschung*, 2000. **50**(2):129-34.
296. Fenton, C., G.M. Keating, A.J. Wagstaff: **Valdecoxib: a review of its use in the management of osteoarthritis, rheumatoid arthritis, dysmenorrhoea and acute pain**. *Drugs*, 2004. **64**(11):1231-61.
297. Flesch, G., P. Muller, P. Lloyd: **Absolute bioavailability and pharmacokinetics of valsartan, an angiotensin II receptor antagonist, in man**. *Eur J Clin Pharmacol*, 1997. **52**(2):115-20.
298. Keating, G.M., L.J. Scott: **Vardenafil: a review of its use in erectile dysfunction**. *Drugs*, 2003. **63**(23):2673-703.
299. Pisani, F., A. Fazio, E. Spina, C. Artesi, B. Pisani, M. Russo, R. Trio, E. Perucca: **Pharmacokinetics of the antidepressant drug viloxazine in normal subjects and in epileptic patients receiving chronic anticonvulsant treatment**. *Psychopharmacology (Berl)*, 1986. **90**(3):295-8.
300. Vandel, B., S. Vandel, J.M. Jounet, D. Blum: **Pharmacokinetics of viloxazine hydrochloride in man**. *Eur J Drug Metab Pharmacokinet*, 1982. **7**(1):65-8.
301. Leveque, D., F. Jehl: **Clinical pharmacokinetics of vinorelbine**. *Clin Pharmacokinet*, 1996. **31**(3):184-97.

302. Rowinsky, E.K., D.A. Noe, D.L. Trump, E.P. Winer, V.S. Lucas, W.A. Wargin, J.A. Hohneker, B. Lubejko, S.E. Sartorius, D.S. Ettinger, et al.: **Pharmacokinetic, bioavailability, and feasibility study of oral vinorelbine in patients with solid tumors**. *J Clin Oncol*, 1994. **12**(9):1754-63.
303. Fourtillan, J.B., S. Bouquet, J. Girault, M.A. Lefebvre, C. Maulet, P. Courtois: **Pharmacokinetics and bioavailability of viqualine, a new antidepressant**. *Eur J Drug Metab Pharmacokinet*, 1985. **10**(1):3-10.
304. Muller, F.O., J.M. Steyn, H.K. Hundt, H.G. Luus: **Warfarin bio-availability. A comparison of 4 products**. *S Afr Med J*, 1988. **74**(11):566-7.
305. Trenk, D., W. Mohrke, L. Warth, E. Jahnchen: **Determination of the interaction of 3S-hydroxy-10,11-dihydroquinidine on the pharmacokinetics and pharmacodynamics of warfarin**. *Arzneimittelforschung*, 1993. **43**(8):836-41.
306. Bastain, W., M.J. Boyce, L.E. Stafford, P.B. Morton, D.A. Clarke, H.F. Marlow: **Pharmacokinetics of xamoterol after intravenous and oral administration to volunteers**. *Eur J Clin Pharmacol*, 1988. **34**(5):469-73.
307. Sorensen, E.V., O. Faergeman, M. Day, W. Bastain: **Pharmacokinetics of xamoterol after intravenous and oral administration to patients with chronic heart failure**. *Eur J Clin Pharmacol*, 1988. **35**(2):183-5.
308. Taylor, I.W., T. Taylor, I. James, G. Doyle, G. Dorf, A. Darragh, L.F. Chasseaud: **Pharmacokinetics of the anti-inflammatory drug ximoprofen in healthy subjects and in disease states**. *Eur J Clin Pharmacol*, 1991. **40**(1):101-6.
309. Taylor, I.W., L.F. Chasseaud, T. Taylor, I. James, G. Dorf, A. Darragh: **Pharmacokinetics of the anti-inflammatory drug ximoprofen in healthy young and elderly subjects: comparison with elderly rheumatic patients**. *Br J Clin Pharmacol*, 1991. **32**(2):242-5.
310. Le Corre, P., G. Dollo, F. Chevanne, R. Le Verge: **Biopharmaceutics and metabolism of yohimbine in humans**. *Eur J Pharm Sci*, 1999. **9**(1):79-84.
311. Dekhuijzen, P.N., P.P. Koopmans: **Pharmacokinetic profile of zafirlukast**. *Clin Pharmacokinet*, 2002. **41**(2):105-14.
312. Adams, J.M., M.J. Shelton, R.G. Hewitt, M. DeRemer, R. DiFrancesco, T.H. Grasela, G.D. Morse: **Zalcitabine population pharmacokinetics: application of radioimmunoassay**. *Antimicrob Agents Chemother*, 1998. **42**(2):409-13.
313. Vanhove, G.F., H. Kastrissios, J.M. Gries, D. Verotta, K. Park, A.C. Collier, K. Squires, L.B. Sheiner, T.F. Blaschke: **Pharmacokinetics of saquinavir, zidovudine, and zalcitabine in combination therapy**. *Antimicrob Agents Chemother*, 1997. **41**(11):2428-32.
314. Cass, L.M., C. Efthymiopoulos, A. Bye: **Pharmacokinetics of zanamivir after intravenous, oral, inhaled or intranasal administration to healthy volunteers**. *Clin Pharmacokinet*, 1999. **36 Suppl 1**:1-11.
315. Blum, M.R., S.H. Liao, S.S. Good, P. de Miranda: **Pharmacokinetics and bioavailability of zidovudine in humans**. *Am J Med*, 1988. **85**(2A):189-94.
316. Wong, S.L., W.M. Awni, J.H. Cavanaugh, T. el-Shourbagy, C.S. Locke, L.M. Dube: **The pharmacokinetics of single oral doses of zileuton 200 to 800mg, its enantiomers, and its metabolites, in normal healthy volunteers**. *Clin Pharmacokinet*, 1995. **29 Suppl 2**:9-21.

317. Salva, P., J. Costa: **Clinical pharmacokinetics and pharmacodynamics of zolpidem. Therapeutic implications**. *Clin Pharmacokinet*, 1995. **29**(3):142-53.
318. Kochak, G.M., J.G. Page, R.A. Buchanan, R. Peters, C.S. Padgett: **Steady-state pharmacokinetics of zonisamide, an antiepileptic agent for treatment of refractory complex partial seizures**. *J Clin Pharmacol*, 1998. **38**(2):166-71.
