# Supplementary material for: Structure-activity models of oral clearance, cytotoxicity, and LD50: a screen for promising anticancer compounds
Source: BMC Pharmacol. 2008 Jun 13;8:12. doi: 10.1186/1471-2210-8-12 (PMC2442056; doi:10.1186/1471-2210-8-12)
Supplement: Additional File 3 — A short mathematical explanation of the KMLA algorithm. A short mathematical explanation of the KMLA algorithm. [file 1471-2210-8-12-S3.doc]

**Additional File 3: A mathematical summary of the KMLA algorithm**

The Kernel Multitask Latent Analysis algorithm [1, 2] is used here with minor changes. Briefly, KMLA uses a kernel function to transform the feature space into a symmetric, positive-definite similarity matrix. Learning occurs via a PLS-like algorithm on the kernel matrix, rather than on the original features. Denote the original feature matrix by $\mathbf{X}\in R^{n\times m}$ and responses by $\mathbf{Y}\in R^{n\times k}$ for *k* tasks. Let a single subscript denote a column of a matrix (e.g., $\mathbf{Y}_{g}$) or a single entry of a row vector. Let the superscript *T* denote transpose, denote the identity matrix as $\mathbf{I}$, and let $[\mathbf{A},\mathbf{B}]$ denote the concatenation of matrices $\mathbf{A}$ and $\mathbf{B}$. The algorithm consists of applying a kernel function to $\mathbf{X}$, thereby creating a kernel matrix $\mathbf{K}\in R^{nxn}$. Next, columns *i*=1,2,...*z* of a matrix of linear orthogonal latent variables, $\mathbf{T}\in R^{n\times z}$, are iteratively generated from $\mathbf{K}$, with $z\ll n$. The goal is to generate $\mathbf{T}$ in such a way that it is a linear projection of $\mathbf{K}$ into a reduced subspace and the loss function $L=\sum_{g=1}^{k} \sum_{i=1}^{n} \delta_{i,g}f_{YF}\left(\mathbf{Y}_{i,g},\mathbf{F}_{i,g}\right)$ is minimized. Here, $\mathbf{F}=\mathbf{TC}$, where $\mathbf{F}\in R^{n,k}$ is a matrix of predicted values, $\mathbf{C}\in R^{z,k}$ is a matrix of coefficients, and $\delta_{i,g}=0$ if $\mathbf{Y}_{i,g}$is missing and $\delta_{i,g}=1$ otherwise.

The loss function can vary between tasks. For linear regression on task *g*, $f_{YF}=\left(\mathbf{Y}_{i,g}-\mathbf{F}_{i,g}\right)^{2}$. Other loss functions could be used if desired. For binary classification on task *g*, targets are labeled as +1 and –1 and an exponential loss function is used, $f_{YF}=\gamma_{i}\exp\left(-\mathbf{Y}_{i,g}\mathbf{F}_{i,g}\right)$. Weights $\gamma_{i}$ allow cost-sensitive learning and can be based on relative frequency of the positive and negative labels. After learning in the subspace is completed, the matrix of PLS coefficients, $\mathbf{C}$, is transformed to a matrix of kernel coefficients, $\mathbf{B}$, such that predictions can be calculated as $\mathbf{F}=\mathbf{\mu}+\mathbf{KB}$, where $\mathbf{\mu}$ is a vector of coefficients for a constant hypothesis. Details of the algorithm are given below.

*Algorithm 1:*

1. Compute the vector $\mathbf{\mu}\in R^{1\times k}$ of optimal coefficients for a constant hypothesis. For the linear regression case, $\mathbf{\mu}_{g}=\bar{\mathbf{Y}}_{g}$ for *g*=1,2...*k*. For binary classification, $\mathbf{\mu}_{g}=\frac{1}{2}\log\left(\frac{\sum_{i\in C^{+}} \gamma_{i}}{\sum_{i\in C^{-}} \gamma_{i}}\right)$ for *g*=1,2...*k*, where $C^{+}$is the set of positive label points, and $C^{-}$is the set of negative label points.
2. Let $\mathbf{D}\in R^{n\times k}$ be a matrix of gradients of *L*. For linear regression on task *g* and a constant hypothesis, $\mathbf{D}_{g}=\mathbf{Y}_{g}-\mathbf{\mu}_{g}$. For classification, $\mathbf{D}_{g}=-\gamma\exp(-\mathbf{Y}_{g}\mathbf{u}_{g})\mathbf{Y}_{g}$.
3. Let $\mathbf{K}^{0}=k(\mathbf{X},\mathbf{X})$ be the initial kernel computed from the kernel function *k*. For a linear kernel, $\mathbf{K}^{0}=\mathbf{XX}^{T}$. Let $\mathbf{K}^{1}=(\mathbf{I}-\frac{1}{n}\mathbf{ee}^{T})\mathbf{K}^{0}(\mathbf{I}-\frac{1}{n}\mathbf{ee}^{T})$ be the centered kernel, where $\mathbf{e}$ is a $n\times1$vector of ones.
4. Let $\mathbf{T}$,$\mathbf{U}$, and$\mathbf{H}$ be empty matrices and let $\mathbf{A}$ be a $n\times n$ identity matrix.
5. For *q*=1 to *z*:
   1. Compute the weight vector $\mathbf{s}\in R^{1\times k}$. For task *g*: $\mathbf{s}_{g}=1-\frac{\left\|\mathbf{D}_{g}\right\|}{\sum_{j=1}^{z} \left\|\mathbf{D}_{j}\right\|}$
   2. Compute the latent factor: $\mathbf{t}^{q}=\mathbf{K}^{q}\mathbf{Ds}^{T}$; $\mathbf{t}^{q}\leftarrow\frac{\mathbf{t}^{q}}{\left\|\mathbf{t}^{q}\right\|}$; $\mathbf{T}=\left[\mathbf{T},\mathbf{t}^{q}\right]$
   3. Compute $\mathbf{u}^{q}=\mathbf{Ds}^{T}$; $\mathbf{U}=\left[\mathbf{U},\mathbf{u}^{q}\right]$
   4. Deflate the kernel matrix: $\mathbf{K}^{q+1}=(\mathbf{I}-\mathbf{t}^{q}\mathbf{t}^{q^{T}})\mathbf{K}^{q}(\mathbf{I}-\mathbf{t}^{q}\mathbf{t}^{q^{T}})$
   5. Compute the function: $(\mathbf{\mu}_{g},\mathbf{C}_{g})=\mathop{\mathrm{argmin}}\limits_{(\mathbf{\mu}_{g},\mathbf{C}_{g})} L(\mathbf{Y}_{g},\mathbf{\mu}_{g}\mathbf{e}+\mathbf{TC}_{g})$ for each task *g*.
   6. Compute the matrix of gradients: $\mathbf{D}=\frac{\partial L}{\partial\left(\begin{matrix} \mathbf{\mu}_{g} \\ \mathbf{C}_{g} \end{matrix}\right)}$ for task *g*.
   7. If q>1, let $\mathbf{A}=\mathbf{A}(\mathbf{I}-\mathbf{t}^{q-1}\mathbf{t}^{q-1^{T}})$.
   8. Compute $\mathbf{H}=\left[\mathbf{H},\mathbf{AU}_{q}\right]$.
   9. Let $\mathbf{B}$ be an empty matrix.
   10. For *p*=1 to *k*:
       1. Compute $\mathbf{B}_{p}=\mathbf{H}\left(\mathbf{T}^{T}\mathbf{K}^{1}\mathbf{H}\right)^{-1}\mathbf{C}$
   11. The final prediction using *q* latent variables is $\mathbf{F}^{q}=\mathbf{\mu}+\mathbf{K}^{1}\mathbf{B}$. In the case of classification on task *g*, a cutoff value, $\psi$ (default equal to zero) is used to separate the two classes.
   12. For predictions on new data $\mathbf{X}_{new}\in R^{r\times m}$, compute the kernel $\mathbf{K}_{new}^{0}=k(\mathbf{X},\mathbf{X}_{new})$ and center it: $\mathbf{K}_{new}^{1}=(\mathbf{K}_{new}^{0}-\frac{1}{n}\mathbf{e}_{new}\mathbf{e}^{T}\mathbf{K}^{0})(\mathbf{I}-\frac{1}{n}\mathbf{ee}^{T})$, where $\mathbf{e}_{new}$ is a $r\times1$vector of ones. Predictions using *q* latent variables are calculated as $\mathbf{F}_{new}^{q}=\mathbf{\mu}+\mathbf{K}_{new}^{1}\mathbf{B}$. Again, a cutoff value is used for separation into binary classes in the case of classification.

Algorithm 1 contains two changes from the one proposed by Xiang and Bennett [2]. First, the original algorithm did not specify how the weight vector $\mathbf{s}$ was to be calculated in Step 5a. Here weights are calculated based on the norms of the column vectors of the gradient matrix $\mathbf{D}$. This is to compensate for large relative differences in gradient magnitudes that can occur for columns of $\mathbf{D}$ when some tasks employ linear regression and others employ classification, or when large differences in gradient magnitudes exist for other reasons.

The second change relates to the way in which the matrix of kernel coefficients $\mathbf{B}$ is calculated. Steps 5g to 5j were developed as a substitute to the method originally proposed. In order to make predictions using the original centered kernel matrix, the matrix of latent feature coefficients, $\mathbf{C}$, must be transformed to a matrix of kernel coefficients, $\mathbf{B}$. To see how this can be accomplished, first consider a non-kernel PLS-like regression algorithm [1] where one task is modeled.

*Algorithm 2:*

1. Calculate $\mathbf{\mu}_{g}$ as in Algorithm 1.
2. Compute $\mathbf{D}$ and $\mathbf{U}$ as in Algorithm 1. Let $\mathbf{W}$ and $\mathbf{P}$ be empty matrices.
3. For *q*=1 to *z*:
   1. Calculate $\mathbf{w}^{q}=\mathbf{X}^{q^{T}}\mathbf{u}^{q}$; $\mathbf{W}=\left[\mathbf{W},\mathbf{w}^{q}\right]$.
   2. Similar to Algorithm 1, calculate $\mathbf{t}^{q}=\mathbf{X}^{q}\mathbf{w}^{q}$; $\mathbf{t}^{q}\leftarrow\frac{\mathbf{t}^{q}}{\left\|\mathbf{t}^{q}\right\|}$; $\mathbf{T}=\left[\mathbf{T},\mathbf{t}^{q}\right]$.
   3. Deflate the data matrix: $\mathbf{p}^{q}=\mathbf{X}^{q^{T}}\mathbf{t}^{q}$; $\mathbf{X}^{q+1}=\mathbf{X}^{q}-\mathbf{t}^{q}\mathbf{p}^{q^{T}}$; $\mathbf{P}=\left[\mathbf{P},\mathbf{p}^{q}\right]$.
   4. Compute the function: $(\mathbf{\mu}_{g},\mathbf{C}_{g})=\mathop{\mathrm{argmin}}\limits_{(\mathbf{\mu}_{g},\mathbf{C}_{g})} L(\mathbf{Y}_{g},\mathbf{\mu}_{g}\mathbf{e}+\mathbf{TC}_{g})$.
   5. Compute $\mathbf{D}$ and $\mathbf{U}$.
   6. Compute $\mathbf{F}=\mathbf{X}^{1}\mathbf{g}$ using the centered data matrix, $\mathbf{X}^{1}$, as described below.

Because of orthogonal columns, $\mathbf{TT}^{T}=\mathbf{I}$, and so $\mathbf{P}=\mathbf{X}^{1^{T}}\mathbf{T}\to\mathbf{PT}^{T}=\mathbf{X}^{1^{T}}$. Therefore:

$$\begin{matrix} \mathbf{X}^{1}\mathbf{W}=\mathbf{X}^{1}\mathbf{W} \\ \mathbf{TP}^{T}\mathbf{W}=\mathbf{X}^{1}\mathbf{W} \\ \mathbf{T}=\mathbf{X}^{1}\mathbf{W}\left(\mathbf{P}^{T}\mathbf{W}\right)^{-1}. \end{matrix}$$

Let $\mathbf{g}=\mathbf{W}\left(\mathbf{P}^{T}\mathbf{W}\right)^{-1}\mathbf{C}$ so that $\mathbf{F}=\mathbf{TC}=\mathbf{X}^{1}\mathbf{g}$.

Algorithm 2 can be turned into a kernel version similar to Algorithm 1 by noting that

$$\begin{matrix} \mathbf{F}=\mathbf{TC} \\ =\mathbf{X}^{1}\mathbf{W}\left(\mathbf{P}^{T}\mathbf{W}\right)^{-1}\mathbf{C} \\ =\mathbf{X}^{1}\mathbf{X}^{1^{T}}\mathbf{U}\left(\mathbf{P}^{T}\mathbf{W}\right)^{-1}\mathbf{C} \\ =k(\mathbf{X}^{1},\mathbf{X}^{1})\mathbf{U}\left(\mathbf{P}^{T}\mathbf{W}\right)^{-1}\mathbf{C} \end{matrix}$$

for a linear kernel. Note also that $\mathbf{t}^{q}=\mathbf{X}^{q}\mathbf{w}^{q}=\mathbf{X}^{q}\mathbf{X}^{q^{T}}\mathbf{u}^{q}\to k(\mathbf{X},\mathbf{X})\mathbf{u}^{q}$ for a linear kernel.

Unlike Algorithm 1, however, where the kernel matrix is deflated, the data matrix is deflated in Algorithm 2 (step 3.c). The deflation step depends on $\mathbf{t}^{q}$ (step 3.b), which depends on $\mathbf{w}^{q}$. But in a kernel version, $\mathbf{W}$ is not explicitly calculated. Therefore, to use a kernel version a new expression for $\mathbf{X}^{1}\mathbf{g}$ is needed. Because $\mathbf{w}^{q}=\mathbf{X}^{q^{T}}\mathbf{u}^{q}$ contains the deflated data matrix, $\mathbf{g}$ must take deflation into account. Note that

$$\begin{matrix} \mathbf{X}^{2}=\mathbf{X}^{1}(\mathbf{I}-\mathbf{t}^{1}\mathbf{t}^{1^{T}}) \\ \mathbf{X}^{3}=\mathbf{X}^{2}(\mathbf{I}-\mathbf{t}^{2}\mathbf{t}^{2^{T}})=\mathbf{X}^{1}(\mathbf{I}-\mathbf{t}^{1}\mathbf{t}^{1^{T}})(\mathbf{I}-\mathbf{t}^{2}\mathbf{t}^{2^{T}}) \\ \mathbf{X}^{4}=\mathbf{X}^{1}(\mathbf{I}-\mathbf{t}^{1}\mathbf{t}^{1^{T}})(\mathbf{I}-\mathbf{t}^{2}\mathbf{t}^{2^{T}})(\mathbf{I}-\mathbf{t}^{3}\mathbf{t}^{3^{T}}) \end{matrix}$$

and so on. Thus, to create a kernel version, let $\mathbf{A}=\mathbf{A}(\mathbf{I}-\mathbf{t}^{q-1}\mathbf{t}^{q-1^{T}})$ for q>1 (see step 5.g of Algorithm 1). Then:

$$\begin{matrix} \mathbf{X}^{1}\mathbf{g}=\mathbf{W}\left(\mathbf{P}^{T}\mathbf{W}\right)^{-1}\mathbf{C} \\ =\frac{\mathbf{X}^{1}\mathbf{X}^{1^{T}}\mathbf{AU}}{\left\|\mathbf{X}^{1^{T}}\mathbf{AU}\right\|}\left(\frac{\mathbf{T}^{T}\mathbf{X}^{1}\mathbf{X}^{1^{T}}\mathbf{AU}}{\left\|\mathbf{X}^{1^{T}}\mathbf{AU}\right\|}\right)^{-1}\mathbf{C} \\ =\mathbf{X}^{1}\mathbf{X}^{1^{T}}\mathbf{AU}\left(\mathbf{T}^{T}\mathbf{X}^{1}\mathbf{X}^{1^{T}}\mathbf{AU}\right)^{-1}\mathbf{C} \\ =k(\mathbf{X}^{1},\mathbf{X}^{1})\mathbf{AU}\left(\mathbf{T}^{T}k(\mathbf{X}^{1},\mathbf{X}^{1})\mathbf{AU}\right)^{-1}\mathbf{C} \end{matrix}$$

The expression for $\mathbf{B}$ is then $\mathbf{AU}\left(\mathbf{T}^{T}\mathbf{K}^{1}\mathbf{AU}\right)^{-1}\mathbf{C}$, which is what we wanted to derive.

To choose an optimal cutoff value, $\psi$, for classifying predictions (step 5.k of Algorithm 1), a modification of the correct classification rate was used as a fitness function. Denote by *a* the fraction of positive labels predicted correctly for a given $\psi$, and denote by *b* the fraction of negative labels predicted correctly. Calculate the score, $s_{\psi}$ as follows: $s_{\psi}=a+b+c$, where $c=0$ if $\min(a,b)>0.65$ and $c=\text{abs}(a-b)$ otherwise. This modification penalizes values of $\psi$ that lead to low values of *a* or *b* and/or a large difference between *a* and *b*.

**References**

1. Momma M, Bennett K: **Constructing Orthogonal Latent Features for Arbitrary Loss**, in *Feature extraction: foundations and applications*, I. Guyon, et al., Editors. 2007, Springer Berlin Heidelberg: New York, NY.

2. Xiang Z, Bennett K: **Inductive transfer using kernel multitask latent analysis.** http://iitrl.acadiau.ca/itws05/Papers/ITWS17-XiangBennett_REV.pdf.
